# Physiological and morphological responses of different spring barley genotypes to water deficit and associated QTLs

**DOI:** 10.1371/journal.pone.0237834

**Published:** 2020-08-27

**Authors:** Dany Moualeu-Ngangué, Christoph Dolch, Michael Schneider, Jens Léon, Ralf Uptmoor, Hartmut Stützel

**Affiliations:** 1 Institute of Horticultural Production Systems, Leibniz University Hannover, Hannover, Germany; 2 Chair of Plant Breeding, Institute of Crop Science and Resource Conservation, University of Bonn, Bonn, Germany; 3 Department of Agronomy, University of Rostock, Rostock, Germany; New South Wales Department of Primary Industries, AUSTRALIA

## Abstract

Water deficit is one of the major limitations to food production worldwide and most climate change scenarios predict an aggravation of the situation. To face the expected increase in drought stress in the coming years, breeders are working to elucidate the genetic control of barley growth and productivity traits under water deficit. Barley is known as a relatively drought tolerant crop and genetic variability was observed for drought tolerance traits. The objectives of the present study were the quantification of morphological and physiological responses in a collection of 209 spring barley genotypes to drought stress, and the genetic analysis by genome-wide association study to find quantitative trait loci (QTL) and the allele contributions for each of the investigated traits. In six pot experiments, 209 spring barley genotypes were grown under a well-watered and water-limited regime. Stress phases were initiated individually for each genotype at the beginning of tillering and spiking for the vegetative- and the generative stage experiments, respectively, and terminated when the transpiration rates of stress treatments reached 10% of the well-watered control. After the stress phase, a total of 42 productivity related traits such as the dry matter of plant organs, tiller number, leaf length, leaf area, amount of water soluble carbohydrates in the stems, proline content in leaves and osmotic adjustment of corresponding well-watered and stressed plants were analysed, and QTL analyses were performed to find marker-trait associations. Significant water deficit effects were observed for almost all traits and significant genotype x treatment interactions (GxT) were observed for 37 phenotypic traits. Genome-wide association studies (GWAS) revealed 77 significant loci associated with 16 phenotypic traits during the vegetative stage experiment and a total of 85 significant loci associated with 13 phenotypic traits during the generative stage experiment for traits such as leaf area, number of green leaves, grain yield, harvest index and stem length. For traits with significant GxT interactions, genotypic differences for relative values were analysed using one way ANOVA. More than 110 loci for GxT interaction were found for 17 phenotypic traits explaining in many cases more than 50% of the genetic variance.

## Introduction

Water deficit is one of the most significant yield-reducing factors worldwide, [1] and agriculture is globally a major water consumer worldwide [2]. Most climate change scenarios suggest an increase in temperature, which will lead to an increase of aridity and water scarcity in many regions of the world [3, 4]. At the same time, the increase of the world population leads to an increase of water and food demands [5]. Risks and consequences of climate change as well as the need for improved cultivars to face water limitations were addressed since the last century [6, 7]. Genetic variability for plant variety improvement is one of the most important factors for increasing the food production in the 21^st^ century. The main requirement for breeders and geneticists to face stressors like water deficit is a wide gene pool with stress tolerance genes [8].

Plant response strategies against drought stress can be distinguished into escapement, avoidance and tolerance strategies [9, 10] and were grouped into short and long term responses. One of the first observed responses to drought stress is stomatal closure to limit the water loss through transpiration [11] leading to a reduction of transpiration [12]. Because the exchange of carbon dioxide (ingress) and water vapour (egress) are regulated through the stomata, transpiration is closely related to biomass accumulation [13–16]. As long-term response to drought stress, plants were observed to reduce their transpiration area in terms of leaf area [11]. Transpiration efficiency (TE), defined as the ratio of above-ground biomass produced per unit transpired water [17–19] at plant level is an important parameter quantifying drought tolerance in terms of resource economics [20]. TE is an indicator of the effective use of the limited water supply, which is needed for yield production. Therefore, improving TE may increase yield in water limited environments [21]. High TE is often associated with drought resistance and discussed as target of breeding programs for crop improvement in water-limited environments [9, 13]. Water deficit may lead to a general decrease of leaf area and dry weight and an increase in root growth [11, 22]. During the grain filling period of barley, drought stress leads to a reduction of the number of fertile spikes per plant and a decrease of the total number of tillers per plant [23]. Plants also acclimate under drought stress by accelerating their development in order to shorten the growth period [24, 25] leading to an earlier ear appearance or decreasing the grain-filling duration of barley, which results in a maturation of the crop [23]. This adaptation strategy generally leads to smaller plants with reduced leaf area and an accelerated senescence, which decreases the yield potential. Osmotic adjustment (OA) is the accumulation of osmotically active substances resulting in a decrease of the water potential to maintain water uptake by the plant [26–28]. To osmotically adjust, plants actively accumulate solutes [26, 29, 30] such as proline [31] and water-soluble carbohydrates [32, 33] to reduce the osmotic potential of the cells, which is necessary to maintain cell turgor. Several authors consider OA as an important component of drought resistance, which contributes to the stability of biomass production under intermittent drought stress [30, 30, 34–36].

Barley (*Hordeum vulgare* L.) is known as the most drought tolerant species of all early mature small grain cereals [37] and because of a high genetic variability, barley is well-adapted to different environmental conditions [38, 39]. Detailed genetic maps are already well developed [37] and a wide pool of available genomic resources was established over the past decades [40], making barley an established species for physiological modelling [41] and a good model species to understand drought tolerance mechanisms [36].

Physiological and morphological changes leading to drought tolerance have a molecular genetic base [42]. With the use of molecular makers it is possible to identify and localize the responsible genes for complex quantitative traits like yield, product quality and the physiological response of the plant under water scarcity. Quantitative trait loci (QTLs) can be used by breeders to systematically improve cultivars [43, 44]. QTL analysis is expected to enhance the effectivity of the selection processes [45]. QTL analysis is based on the identification of statistical correlations between a quantitative phenotypic trait and specific genetic makers in the genome [46]. The chromosome regions with the highest impact on a trait can be identified as QTL. Several studies investigated and identified QTL for plant responses to water deficit, e.g. for osmotic adjustment in rice [27] and barley [42, 36, 32], plant water status, water-soluble carbohydrate [32] and relative water content in barley [45], leaf growth of maize [47, 48], flag leaf senescence of wheat [49], drought stress induced leaf senescence in juvenile barley [50], water-use efficiency in barley [21], barley yield components and seed quality under terminal drought [51, 52] and drought tolerance in wild barley [53]. An overview of genetic mapping studies in barley and wheat aimed at the determination of major QTLs affecting drought-associated traits and the final yield characteristics is given [54].

Passioura [20] defined yield or grain dry mass (*W*_g_, g/plant) as

$$W_{g}=W_{t}∙p_{g},$$

where *W*_t_ (g/plant) is the total plant dry mass and *p*_g_ is the harvest index, defined as the ratio of grain yield to the above-ground dry matter. On the other hand, *W*_t_ is can be estimated as

$$W_{t}=T*TE,$$

where T(l/plant) is the total amount of water transpired by the plant and TE is the transpiration efficiency. The difficulty at this level is the estimation of the total amount of water transpired by the plant. T can be calculated as

T=ST*LA,

where ST (l/m^2^/plant) is the specific transpiration, defined as the amount of water used per square meter leaf during the whole growing time and LA (m^2^/plant) the total leaf area. The specific transpiration was reported to increase significantly with increasing vapour pressure deficit and daily total light integral [55]. Therefore, the decrease in yield under water deficit might be due to the change in each of the components defined above.

The aim of the present study was the quantification of the physiological and morphological responses of different spring barley genotypes to water deficit as well as the characterization of the genetic base by GWAS to find QTL for the investigated traits. Therefore, physiological relationships of yield production in relation to water deficit in the vegetative and generative stages were investigated. The results of this study provide information for breeding purposes and can be used to develop genetically-based physiological equations to model plant growth under drought stress conditions *in silico*.

## Material and methods

### Plant material and experimental setup

209 spring barley genotypes belonging to the “barley core collection” [56], obtained from the Leibniz Institute of Plant Genetics and Crop Plant Research (IPK, Gatersleben, Germany) and adapted to Central European weather conditions were investigated. The genotypes cover a wide range of species and origins representing the genetic variability of cultivated barley and the wild species of *Hordeum* (See S1 Fig for the ancestry matrix). Six pot experiments were conducted from 2012 to 2015 in an open-sided screenhouse at the Institute of Horticultural Production Systems, Leibniz Universität Hannover, Germany (52.5° N, 9.7° E). In these experiments, physiological and morphological traits of all genotypes grown in a well-watered control and a water deficit treatments were investigated.

The water deficit treatment was induced by stopping the water supply, which led to a continuously decreasing amount of plant available water in the soil. For each experiment, three seeds were sown in each of the two pots allocated to each genotype (well-watered and water shortage). Pots were 11.8 cm in diameter and 57 cm in height, and filled with loess soil (bulk density: 1.35 g cm^-3^). The soil was passed through a screen to remove large aggregates and mixed before use. The gravimetric water content of a soil sample was analysed and each pot was filled with 8 kg of dry soil. The analysis and calculation methods were similar to those described in [57]. According to a water retention curve for loess [58], around 30.033% of the pore volume can hold plant available water. All pots were irrigated up to 100% water holding capacity by adding 1,780 ml water per pot. Around 1,350 ml water per pot was available for the plants. Depending on the germination ability of each variety, a specific number of seeds per pot were sown directly. After emergence, seedlings were thinned to three plants per pot. Each of them was used for different measurements: one was used for destructive measurements such as OA, one was harvested after the stress treatment and the last one was harvested after the regeneration stage (only in the vegetative experiments). To minimize soil evaporation, pot surfaces were covered with a layer of quartz gravel [59].

In each experiment, the 418 pots (two per genotype) were surrounded by 152 additional pots as border to reduce boundary effects (S2 Fig). All pots were placed close to each other to simulate a crop-like setup. At the beginning of the experiments, all pots were irrigated up to 100% of water holding capacity. The experiments were arranged in a split-plot design with 209 genotypes and two irrigation levels. Six experiments with the same setup were established over the years 2012 to 2015 and used as repetitions (S2 Fig). In experiments 1, 2 and 4 only the vegetative stage of the plants was investigated while in experiments 3, 5 and 6 stress cycles were conducted during the generative stage. In the vegetative stage experiments, the water deficit treatment was initiated for individual genotypes at beginning of tillering (BBCH scale 21) while in the generative stage experiments the water deficit treatment was initiated for individual genotypes at spiking (BBCH scale 51, S2 Table). In both cases, at least two of the three plants per pot should have reached the stage for stress initiation. After stress initiation, only the control pots were further irrigated three times per week until the transpiration rate of the water deficit plants reached 10% of the corresponding well-watered pot [59]. After reaching this threshold the stress period of each genotype ended.

### Measurements and calculations

During the experiments, all pots were weighted three times per week to calculate transpiration and soil water content. Relative transpiration (rTR) was defined as the ratio of transpiration (TR, ml) of the plants under drought stress by the one of the control plants. The percentage of water extracted by the plants in the water deficit treatment (Wex, %) was estimated as the ratio of the difference of soil water content between the beginning of the stress treatment and the end of the stress treatment by the soil water content at the beginning of the experiment. The fully developed flag leaves of the plants reserved for destructive measurements were used for determination of proline contents (PROL, μmol g^-1^ fresh weight) and osmotic adjustment (OA, MPa). When the relative transpiration was around 20% and the flag leaves started to wilt, samples of both the well-watered and water-limited plants’ flag-leaves were taken. Two leaf discs with a diameter of 0.6 cm were punched out, placed in aluminium foil and shock frosted in liquid nitrogen for osmotic potential measurement. Another piece from the same leaf was used to determine the relative leaf water content by weighing directly after cutting to determine its fresh weight (FW, g), then, the sample was soaked in demineralized water under light conditions for 24 hours to measure turgor weight (TW, g). Thereafter, the sample was dried in an oven at 70°C for 24 hours to determine the dry weight (DW, g). The relative water content was calculated according to Bars and Weatherley [60]:

$$RWC=\frac{(FW-DW)*100}{TW-DW}.$$ (1)

The osmotic potential (OP, MPa) was measured with a psychrometer in C-52 chambers (PSYPRO, Wescor Inc., Logan, Utah, USA). OP was multiplied with the RWC to estimate the osmotic potential at full turgor for both the well-watered (OP_100w_) and the water deficit plants (OP_100d_) [36, 61]. OA was estimated as the difference between OP_100d_ and OP_100w_ [62, 36]

$$OA={OP}_{100d}-{OP}_{100w}.$$ (2)

OA and PROL were determined from the same leaves. For PROL determination the sample was shock frosted in liquid nitrogen first and free proline content in the leaves was determined following the method of Bates et al. [63]. After the stress phase, plants were harvested to determine total plant fresh mass (FM, g), number and average length of the tillers (N_t_ and L_t_ (cm), respectively), length of the main stem (Lms, cm), phenological development stage of the main stem [64], dry mass of the ear of the main stem (DMEms, g), ear number (NE) and dry mass of tillers (DMt, g), dry mass of main stem and tiller stalk (DMSms and DMSt, g), number of green (NLG) and senescent leaves of the main stem (NLSms) and tillers (NLSt), leaf length (LL) and leaf area of green leaves (LAGLms) of the main stem and leaf area of green leaves of the tillers (LAGLt). After dividing the plants into single plant organs, they were dried in an oven and weighted to obtain the dry mass (DM).

TE was obtained as ratio of plant dry mass by total plant transpiration and HI was estimated by the ratio of grain yield to above-ground dry matter.

For almost every measured or calculated parameter, relative values were calculated from absolute values of each genotype as the ratio of the value obtained from the plant under drought stress by the one obtained from well-watered plants and the acronyms are summarized in Table 1 (column 4).

**Table 1:** Summary of key traits derived or measured and their abbreviations and units.

Trait	Abbreviations	Unit	Relative value Abbreviations	
(1) Dry mass				
Total plant dry mass	DM	g plant^-1^		rDM
Total ears dry mass	DME	g plant^-1^		rDMEt
Dry mass of the tiller’s ears	DMEt	g plant^-1^		DMEt
Dry mass ear of the main stem	DMEms	g plant^-1^		rDMEms
Total dry mass of green leaves	DMLG	g plant^-1^		rDMLG
Dry mass green leaves of the main stem	DMLGms	g plant^-1^		rDMLGms
Dry mass green leaves of tillers	DMLGt	g plant^-1^		rDMLGt
Dry mass of senescent leaves of the main stem	DMLSms	g plant^-1^		rDMSLms
Dry mass of senescent leaves of the tillers	DMLSt	g plant^-1^		rDMLSt
Total dry mass of the stems	DMS	g plant^-1^		rDMS
Dry mass of the stem on the main stem	DMSms	g plant^-1^		rDMSms
Dry mass of the stems on tillers	DMSt	g plant^-1^		rDMSt
(2) Counts				
Number of green leaves	NLG			rNLG
Number of tillers	Nt			rNt
Number of tiller ears	NEt			rNEt
(3) Morphological traits				
Total leaf area	LA	cm^2^ plant^-1^		rLA
Leaf area of green leaves of tillers	LAt	cm^2^ plant^-1^		rLALGt
Leaf area of green leaves of the main stem	LAms	cm^2^ plant^-1^		rLALGms
Leaf area of the flag leaf of the main stem	LAFms			rLAFms
Length of the main stem	Lms	cm plant^-1^		rLms
Average length of the stems of tillers	Lt	cm plant^-1^		rLt
Length of the flag leaf of the main stem	LFms	cm plant^-1^		rLFms
Specific leaf area of the whole plant	SLA	cm^2^ g^-1^		rSLA
Specific leaf area of tillers	SLAt	cm^2^ g^-1^		rSLAt
Specific leaf area of the main stem	SLAms	cm^2^ g^-1^		rSLAms
(4) Physiological traits				
Proline content	PROL	μmol g^-1^		rPROL
Osmotic potential	OP	Mpa		rOP
Relative leaf water content	RWC	%		rRWC
Transpiration efficiency	TE	g l^-1^		rTE
Harvest index	HI	g g^-1^		rHI
Total transpired water	T	l		rT
Percentage of water extracted by the plants in the water deficit treatment	Wex	%		
Specific transpiration	ST	l m^-2^ plant^-1^		rST
Phenological development stages (0–100)	BBCH			rBBCH

In experiment 6 water soluble carbohydrates (WSC, mg g^-1^) within the stalk were investigated to assess the influence of drought on translocation from stems. WSC are sugars (primary glucose, fructose, sucrose, and fructans), which accumulate in the stalk of cereals around anthesis. They serve as a reservoir for remobilization to the developing grains [65]. Samples were first taken from well-watered plants at the end of heading (BBCH-scale 59) when spikes were fully emerged. According to a pre-test and [65], WSC in the stalk reached their maximum at this stage. Thereafter, the stress period was initiated (at BBCH-scale 60) and at the end of the water deficit treatment, second samples were taken. According to the protocol of Maness [66], stalks were cut into 5 cm long pieces, placed into a forced draft oven at 90°C for 60 to 90 minutes, then samples were transferred into the oven with 70°C for 24 hours. Afterwards the samples were ground with a mixer mill (Retsch MM400 with 50 ml steel beaker with steel ball, Retsch AG, Arzberg, Germany) and WSC were extracted with hot water by using the Anthrone Procedure [67]. The relative WSC (rWSC) content in the stalk was calculated by the ratio of WSC after the stress phase by WSC content before stress, at the end of heading. From rWSC, the percentage amount of WSC translocated from stalk was calculated by

WSCtranslocated[%]=(1−rWSC)*100. (3)

If more WSC were accumulated in the stalk after stress than in the control sample, the value for WSC translocated became negative. Since just measured translocation was of interest, negative values were excluded from the dataset. All traits and their acronyms were summarized in Table 1.

### Data preparation and statistical analysis of phenotypic data

During the experiments, sometimes plant organs were damaged or lost. To reduce missing dry mass data, lost ears of the main stem were estimated by an ear to straw ratio of the corresponding plant or alternatively, the weight of a single ear of a tiller was used to estimate dry mass of the main stem ear. From 29 plants of the first experiment and 2 plants of the second experiment, only the total fresh weight, but no dry mass data was available. The missing total dry weights were estimated by the total fresh weight multiplied by the average dry to fresh mass ratio of the specific irrigation level.

Before performing the statistical analysis and calculating average values, the physiological data of observations were separated into a vegetative and a generative dataset and cleaned for outliers. In fact, after cleaning the total dry mass of the control plants, observations of the relative dry mass were cleaned by removing outliers for each genotype. Total ear dry mass and the total area of green leaves were expected to be negatively affected by drought stress. Therefore, data of observations with a relative value > 2 were removed. The statistical analysis was performed by a linear mixed model and Tukey HSD-test in R-3.2.5 (R Core Team, 2016) using the packages lme4 [68] and agricolae [69].

### Genome-wide association study (GWAS)

6259 SNPs [70] were used for the association study. The success rate of genotyping was higher than 97%, leaving less than 3% of missing data. A genetic map was constructed for these markers and the first three principal components as well as the kinship were calculated. The population structure was investigated by the R package LEA [71] and the linkage information for the population was checked by applying the R package Synbreed [72]. For ladder, we used the physical instead of the genetic position.

The genome-wide association study was performed on the measured values and the relative values (ratio of the stress value by the well-watered value) of different phenotypic traits. For determination of phenotype-genotype associations, we used the SNP marker, population structure and kinship matrix data. The population structure was performed by principal component analysis using the function prcomp in R and kinship matrix were calculated using 6259 polymorphic SNP in the R-package ‘rrBLUP’ [73]. Phenotypic traits of plants under stress and well-watered conditions were used for this investigation separately for vegetative and generative data. Genome-wide association mapping was performed following the GRAMMAR method described by [74]. For the analysis, including the first three principal components and the kinship matrix as co-actors to control for population structure were included to the analysis additionally to the biallelic markers. The methods of the analysis were used and described in detail by Reinert et al. [75] and Naz et al. [76]. We used a linear mixed model to calculate the QTLs as presented below:

$$Y_{ijk}=\mu +M_{i}+T_{j}+M_{i}*T_{j}+L_{k}\;(M_{i})+\varepsilon_{ijk}$$ (4)

where Y_*ijk*_ is the phenotypic value; μ is the general mean; *M*_*i*_ is the fixed effect of *i*-th marker genotype/haplotype; *T*_*j*_ is the random effect of *j*-th treatment; *M*_*i*_ * *T*_*j*_ is the interaction effect of the *i*-th marker with the *j*-th treatment; *L*_*k*_ (*M*_*i*_) is the random effect of *k*-th barley line nested within *i*-th marker genotype/haplotype and ε_*ijk*_ is the residual. To determine QTLs of interest in the genome-wide detection analysis a log of odds (LOD) threshold with *p-*value ≤0.0001 and 1,000 permutations was determined. The QTL-model comprises an iterative multi-locus procedure. Therefore, the most informative SNP (QTL) was set as a fixed factor during each calculation iteration step. All remaining markers were again incorporated in the next iteration round and reanalyzed. The starting point of next calculation round was determined by the result of the previous iteration. *P-*values of significant markers were corrected using probability of false discovery rate (PFDR), implemented in the SAS procedure PROC MULTTEST according to Benjamini & Yekutieli [77]. This procedure was repeated until no marker could be detected, which led to a reduction of significant markers and thereby a reduced number of false positive QTL. SNPs were combined to one joint QTL depending on their estimated (significant) *p*-value from the first iteration of the multi-locus procedure. Therefore, the size of the genetic interval was dependent on the significance value of flanking SNPs. A “leave-20%-out” cross validation procedure was used to increase the validity of all significant SNPs. Every genotype was investigated individually and a QTL had to be significant in both stress and well-watered treatments to be classified as significant QTL for the measured phenotypic traits. A false discovery rate (FDR) smaller than 0.05 and logarithm of the odds (LOD) score greater than or equal to 3.0 are often set as thresholds to declare the presence and correctness of a found QTL [78, 79]. Markers outside this thresholds were dropped from the output files and considered as non-significant. The broad sense heritability was calculated as described in [80] and used in [75].

## Results

### High degree of variability on phenotypic data during both vegetative and generative experiments

Physiological and morphological traits were investigated under well-watered and water deficit conditions during both, the vegetative and the generative stages (S1 Table). Significant genotypic differences were found for all phenotypic traits, indicating a broad variability amongst the genotypes investigated in this study. Interestingly, for the important trait of specific transpiration, no significant genotypic variation was found. Significant water deficit effects were observed for parameters such as total plant dry mass (DM), total leaf area (LA, vegetative experiments), dry mass of green leaves (DMGL, vegetative experiments), proline content (PROL), osmotic potential (OP), relative leaf water content (RWC), the stalk dry mass (DMS, vegetative experiment), specific transpiration rate (ST, vegetative experiments) and the length of the main stem (Lms, vegetative experiments). DM was significantly decreased by the water deficit (Table 2). This might be due to the lower tiller number and an accelerated leaf senescence. However, a higher proline concentration in the stem and a higher leaf OP, which represent typical adaptation strategies under water deficit was measured in the water limited plants. These observations also suggested the existence of tolerance through OA in the barley varieties under study.

**Table 2:** Analysis of variance for genotypic and treatment effects and their interactions on different phenotypic plant traits using a linear mixed model during the both the generative and vegetative stage experiments.

Generative				Vegetative				Generative				Vegetative			
Trait	G	T	G x T	Trait	G	T	G x T	Trait	G	T	G x T	Trait	G	T	G x T
**DM**	***	**	***	**DM**	***	*	***	**DMLG**	***	***	***	**DMLG**	***	**	***
**DME**	***	*	***	**DME**	***	***	***	**DMLSms**	***	*	**	**DMLSms**	***	*	***
**DMEt**	***	*	***	**DMEt**	***	**	***	**DMEms**	***	.	***	**DMEms**	***	*	***
**DMLGt**	***	***	***	**DMLGt**	***	**	***	**DMSt**	***	*	***	**DMSt**	***	*	***
**DMLGms**	***	***	***	**LAt**	***	*	***	**LAt**	***	*	**	**T**	***	*	***
**Lams**	***	***	***	**LAms**	***	*	***	**LA**	***	***	***	**TE**	***	*	***
**NLG**	***	*	***	**NLG**	***	**	***	**Lt**	***	**	***	**ST**		***	**
**Net**	***	*	***	**NEt**	***	***	***	**OP**	***	***	***	**OP**	***	***	***
**BBCH**	***	.	***	**BBCH**	***	***	*	**PROL**	***	**	***	**PROL**	***	**	***
**Nt**	***	.	***	**Nt**	***	*	***	**RWC**	***	*	***	**RWC**	***	***	***
**HI**	***			**HI**	***		***	**SLAt**				**SLAt**			
**Lms**	***			**Lms**	***	**	***	**SLAms**	***	***	***	**SLAms**	***	*	***
**LFms**	***	***	***	**LFms**	***	*	***	**SLA**	***	***	***	**SLA**	***	*	***

Refer to Table 1 for all acronyms and units. G: Genotype, T: Treatment and GxT: genotype x treatment interaction; significance codes

'***' 0.001

'**' 0.01

'*' 0.05; '.' 0.1.

GxT interactions were found for almost all investigated traits, revealing the plasticity of these traits in both generative and vegetative experiments. During the vegetative experiments, GxT interactions were found for all dry matter related traits (DM, WG, DMEt, DMEms, DMLG, DMLSms, DMS and DMSt), physiological traits (PROL and OP) and morphological traits (LA, Lt, Lms, LAFms, LFms, Lms, SLA, ST). The reduced DM production under water deficit in the generative experiment was mainly due to the decrease of DMEt and DMLS suggesting that barley genotypes tended to accelerate senescence of tillers during water scarcity in the generative stage. Genotypic effects and GxT interactions were found for water related parameters such as T, RWC and TE during the vegetative stage experiments. Also during the vegetative stage experiments, genotypic variation and interactions were found for HI suggesting an escapement strategy for some genotypes under water deficit. However, there was no GxT interaction for HI during the generative stage experiment.

### Variability in relative values underlying phenotypic plasticity

High genotypic variation (p < 0.001) was found for functional traits such as relative dry mass of individual plant organs (rDM, rDME, rDMS, rDMLG) during both generative and vegetative stage experiments (S2 Table). Genotypic variation was also found for water related parameters such as rT during the vegetative stage and for physiological traits such as OA, rPROL, rOP during both vegetative and generative stage experiment. Moreover, genotypic effects were found for morphological traits such as leaf areas (rLA), specific leaf area (rSLA) as well as the relative length of the main and tiller stems (rLms, rLt) during both the vegetative and the generative stage experiments. Genotypic variability for the relative leaf area of green leaf rLA revealed a difference in leaf senescence under water deficit.

Genotypic variation was also observed for relative agronomic traits such as rTE in the vegetative stage, rHI, rBBCH (relative phenological stages) and rNLG after the generative and vegetative stage treatments. In the vegetative experiment, rNt was genotype dependent suggesting a clear variability amount genotypes in terms of tillers production under water deficit.

### Significant genotypic variation in phenotypic traits

**Fig 1:**
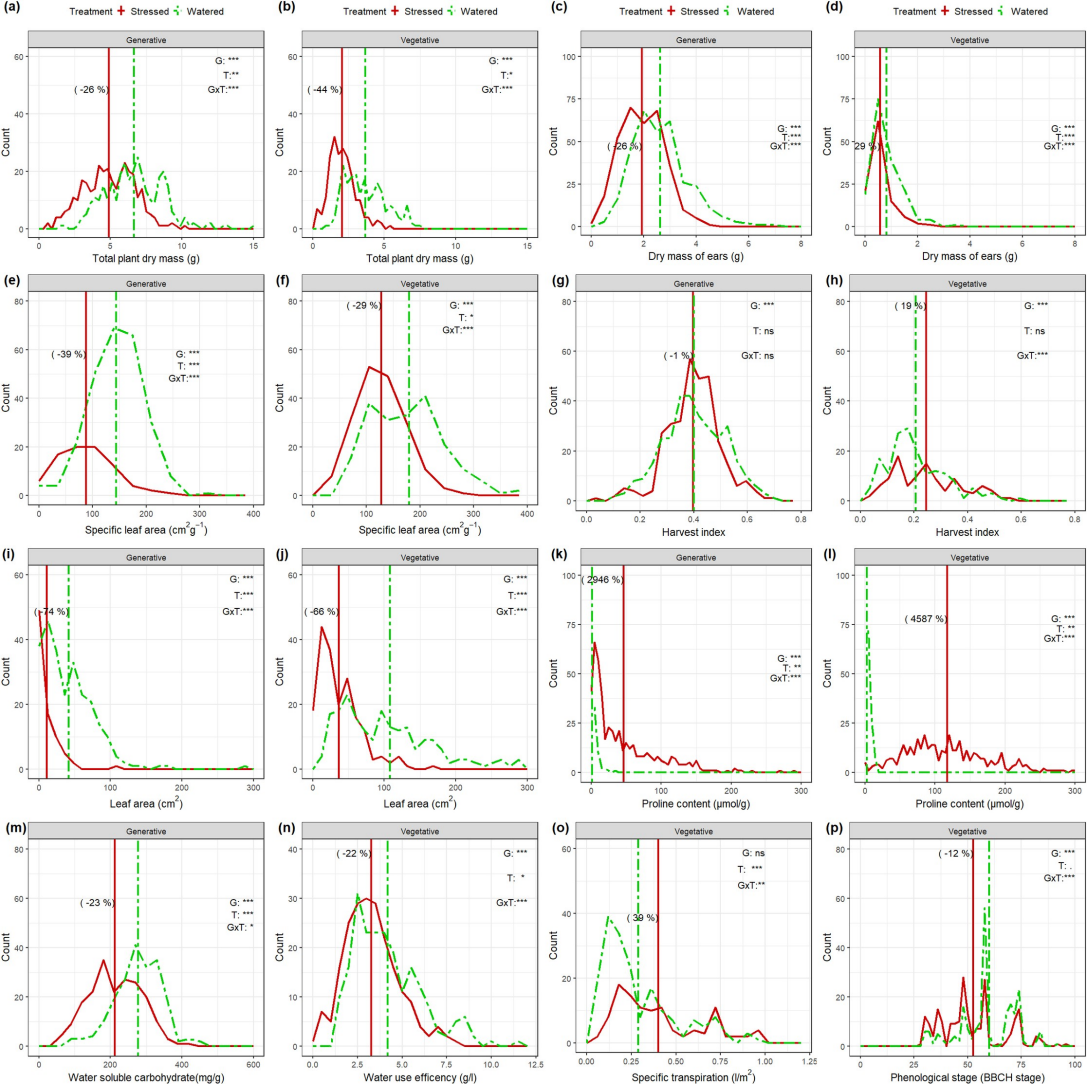
Frequency distributions of physiological and morphological traits comparing the frequency of well-watered control (green dash-dotted lines) and drought stress (red plain lines) during both generative and vegetative stage treatments. The vertical lines in the histograms show population mean values in control (green) and water deficit stress (red) conditions, and values in parentheses represent the percentage change (+, increase;–, decrease) in water-deficit conditions over the control. Levels of significance for genotype (G), treatment (T), and their interaction (GxT) effects from the linear mixed model are given in the graphs (***, P< 0.001; and ns, not significant).

**Fig 2:**
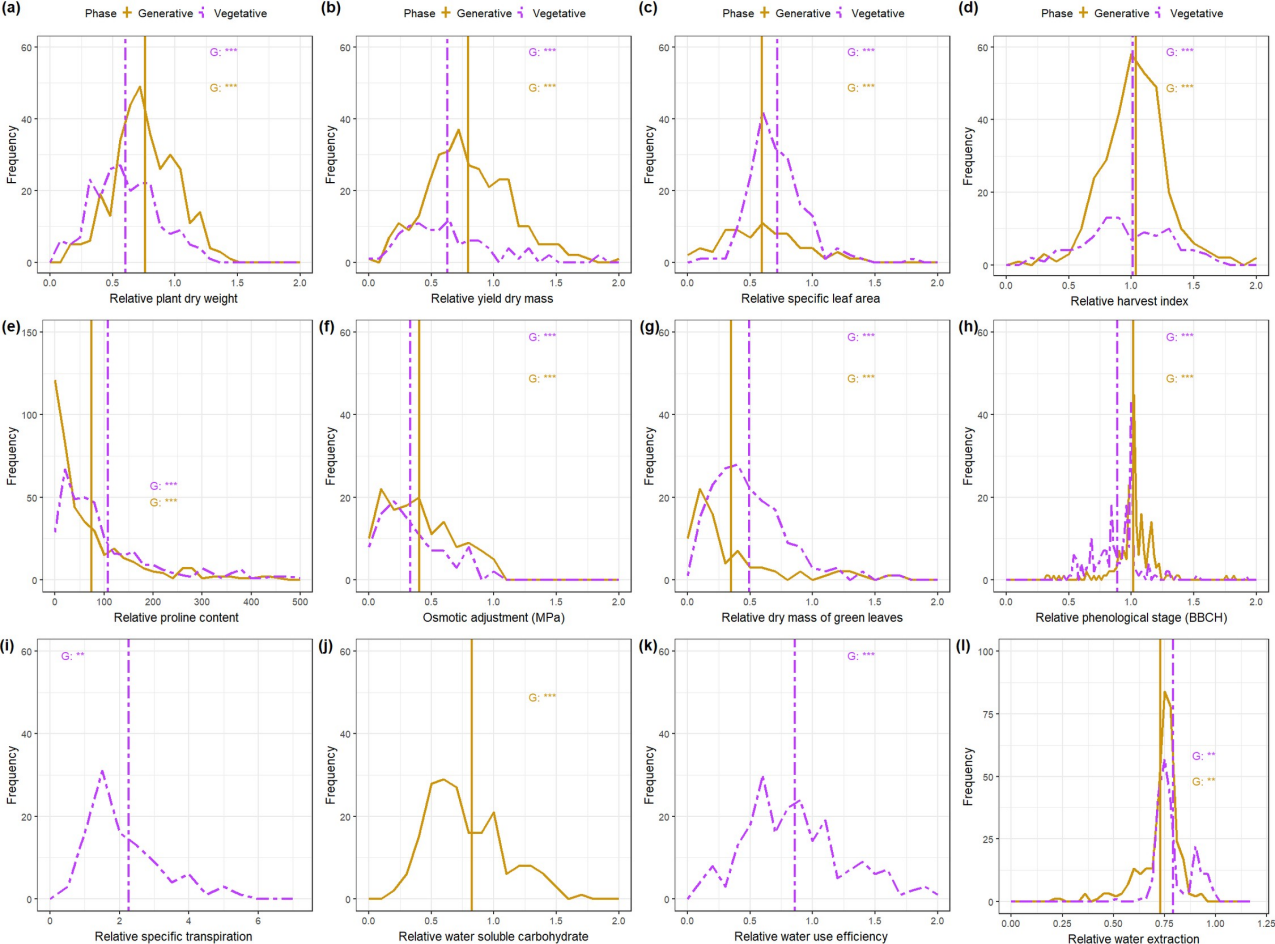
**Frequency distributions of physiological and morphological traits comparing the frequency of relative values during the generative stage (yellow plain lines) and vegetative stage (violet dash-dotted lines) water deficit treatments.** The vertical lines in the histograms show population mean values in control vegetative and generative stage treatments. Levels of significance for genotype (G) from the Tukey test are given in the figure (***, P< 0.001; and ns, not significant).

The distributions of shoot traits with GxE interactions and relative water deficit treatment effects are depicted in Figs 1 and 2, respectively. Drought stress decreased the average total dry mass by 26% in the generative stage experiments and roughly 44% in the vegetative stage experiments, indicating that the water shortage was on average more severe for the plant during the vegetative stage (Fig 1A and 1B). The decrease of DME after the water deficit treatment was roughly 26% during the generative stage (Fig 1C), while water withholding during the vegetative stage lead to a reduction of DME by 29% (Fig 1D).

As expected, effects of water deficit on the number of tillers (N_t_) were only observed at the end of the vegetative stage experiment, not during the generative stage. N_t_ had different distributions among both vegetative and generative experiments. A clear shift of the distributions of water-limited plants compared to the well-watered ones was also observed for DMSt (66% and 34%, Fig 1O and 1E), LA (74%, 66%), SLA (39%, 29%), PROL (Fig 1K and 1L) and TE (22%, Fig 1N). Although water shortage lead to a reduction of these traits, the distributions of few traits were changed. In fact, the distributions for DMSt, LA and DMLG in the vegetative stage were much narrower in the stress treatment than in the control. PROL was highly increased in the water-limited plants both during the vegetative and generative stage experiments for a large number of genotypes (Fig 1K and 1L). Water shortage lead to a significant decay of the number of green leaves and their dry mass. The decay was more effective during the generative stage experiment than in the vegetative one.

Diversity in the responses of the different plant traits to water deficit illustrates the range of the responses and phenotypic plasticity of each trait. Phenotypic plasticity indicators such as rDM, rDME, rLA, rDMt, rDMLG, rSLA, and rTE showed a significant difference for most genotypes between the generative and the vegetative stage experiments (Fig 2; S1 Table). This is indicated by the shift of the distribution to the left (relative values less than 1). However, the distributions of rNt and rHI remained centred at 1, illustrating that the number of tillers, the number tillers with ears, and the harvest index was largely unaffected by water deficit. The percentage of water extracted by each genotype (Wex) covered a wide range in both vegetative and generative stage experiments (Fig 2L). However, an on average higher Wex was obtained during the vegetative stage experiments than during the generative ones, indicating a higher root activity in the vegetative stage.

### Correlations between phenotypic traits under water deficit

**Fig 3:**
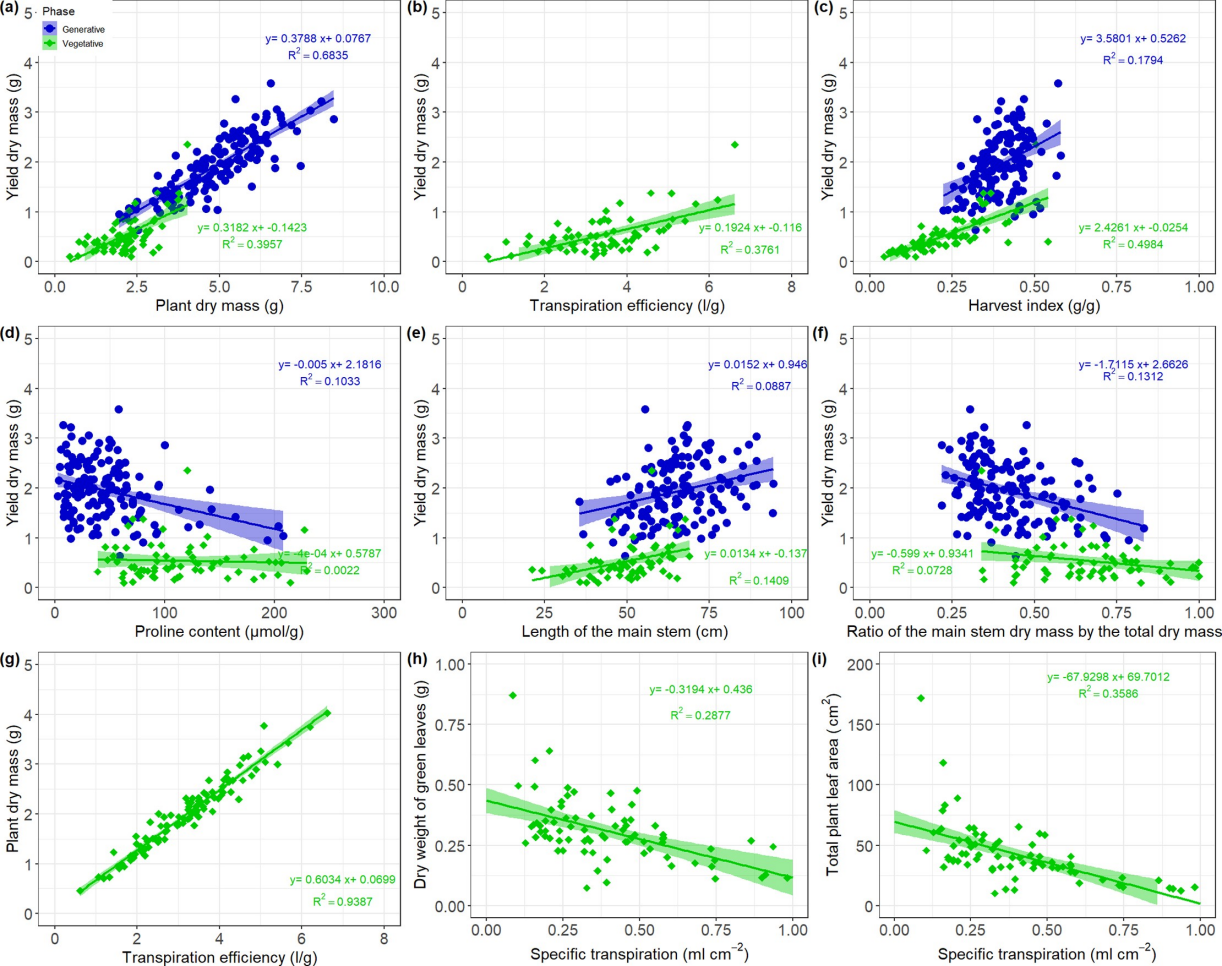
Regression plots between selected phenotypic traits for vegetative (green) and generative (blue) experiments (refer to S3 Fig for the summarized correlation matrix). (a-f) present the correlation of plant dry mass, transpiration efficiency, harvest index, proline content, length of the main stem and the fraction of the main stem dry mass over the total plant dry mass on the yield; (g) plant dry mass and transpiration efficiency; (h-i) specific transpiration and dry weight of green leaves, and total plant dry weight respectively.

Plant phenotypic traits are the results of complex combinations between different mechanisms, which can be explained by correlations. As expected, ears dry mass (DME) correlated well with traits such as DM, HI, TE, PROL (Fig 3A–3D), DMS and DMLS (S3 Fig) during the vegetative and generative stages. No significant correlation with LA or ST was found for DME suggesting a more complex relationship between ST and leaf area with yield.

Although DM had a strong correlation with TE during the vegetative stage experiments, surprisingly there was no correlation with LA, T or any other water related parameter such as ST. The total plant dry weight was therefore mainly explained by the dry mass on individual organs, TE, Lt and Lms (S3 Fig). T had significant negative correlations with the number of tillers and TE, suggesting that genotypes with low number of tillers tended to have a slightly higher total amount of water transpire (S3 Fig). The total leaf area correlated well with traits such as the specific transpiration and the specific leaf area (|r| > 0.45). The specific transpiration also correlated with some other traits such as DMLG, NLG, and SLA suggesting possible co-localisation of genes for these traits. The proline content was slightly negatively correlated with DME (r = -0.38, Fig 3B) during the generative stage experiment suggesting that varieties with high proline content tend to have lower yield under water deficit probably due to the active proline production. OP_100d_ did not correlate with any plant trait during the vegetative experiment, but positively correlated with PROL during the generative stage. TE strongly correlated with DM (r = 0.97), Lms (r = 0.57), Lt (r = 0.53) and Nt (r = 0.37, Fig 3B) in water-limited plants, indicating a high correlation with the whole plant performance.

**Fig 4:**
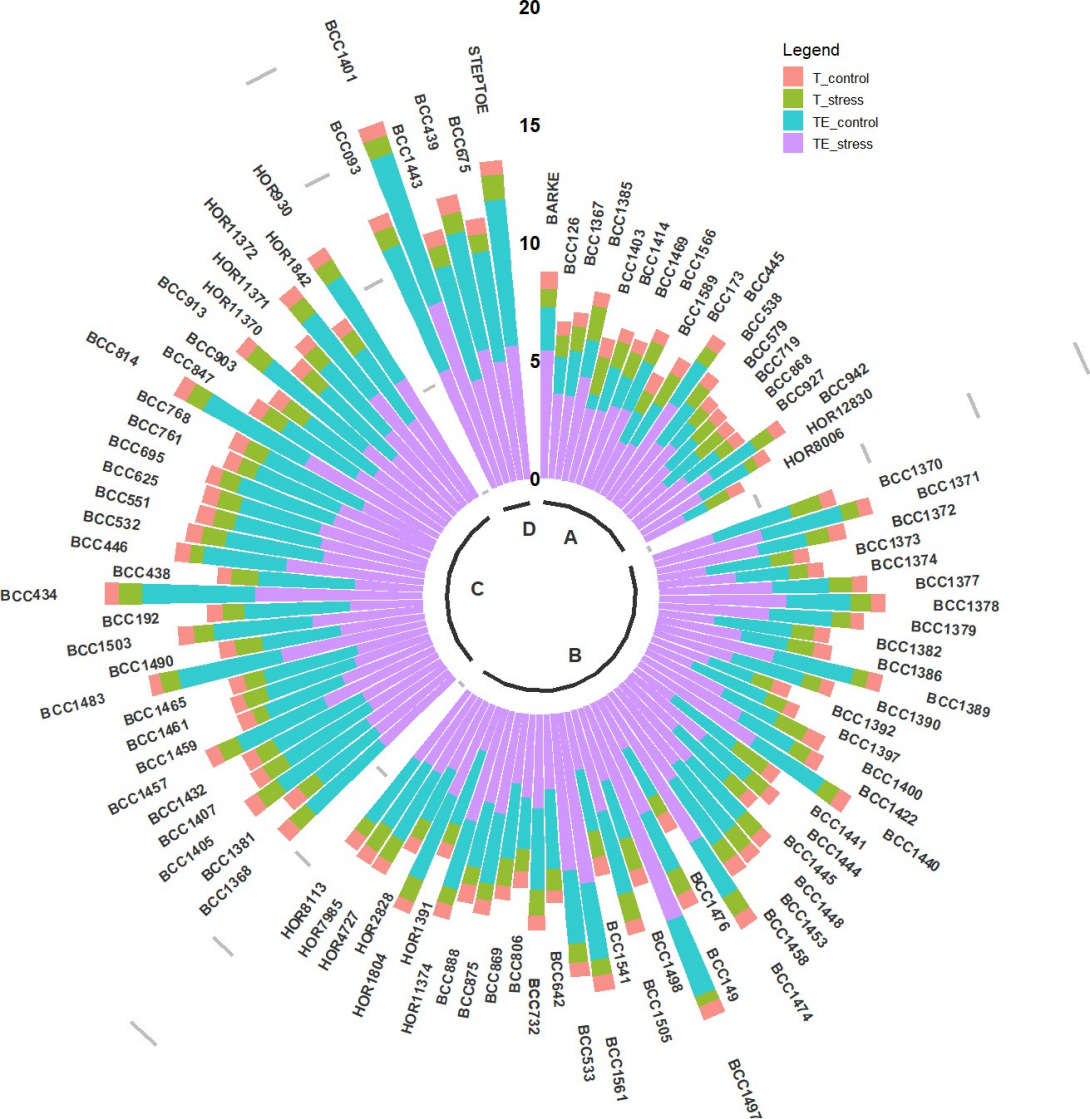
Relationship between the total plant dry mass (DM) under drought stress, the cumulative transpiration (T, l) and the transpiration efficiency (TE, g/l) of different varieties. A, B, C and D represent the varieties specific class of normalized total dry weight in [0, 25[%, [25, 50[%, [50,75 [% and [75,100]% respectively.

In the generative stage experiments, WSC had a weak negative correlation (r < -0.39) with traits such as SLA, LA, Lms or DMLG. Under water deficit, WSC was positively correlated only with the proline content (r = 0.24). However, WSC did not correlate with the dry mass of senescent leaves or any dry weight related trait after the stress treatment. HI correlated negatively with DME and all other dry mass related traits during the vegetative experiments. The reduction of DMt caused by water deficit had a strong negative impact on total plant DM. In fact, DMt was more affected by the stress treatment than DMms. Observing individual genotypes during the vegetative stage experiment revealed that varieties with high DM were also varieties with high TE and T (Fig 4). However, several genotypes presenting a high TE under drought stress such as BCC1497, BCC1474, BBC1561 or BCC1389 for instance, had a low dry mass under stress condition. Some other genotypes classified in the category B (with normalize values between 25% and 50%, Fig 4) increased significantly the TE under water deficit, although they ended having less biomass under drought stress.

### Correlation of relative values

**Fig 5:**
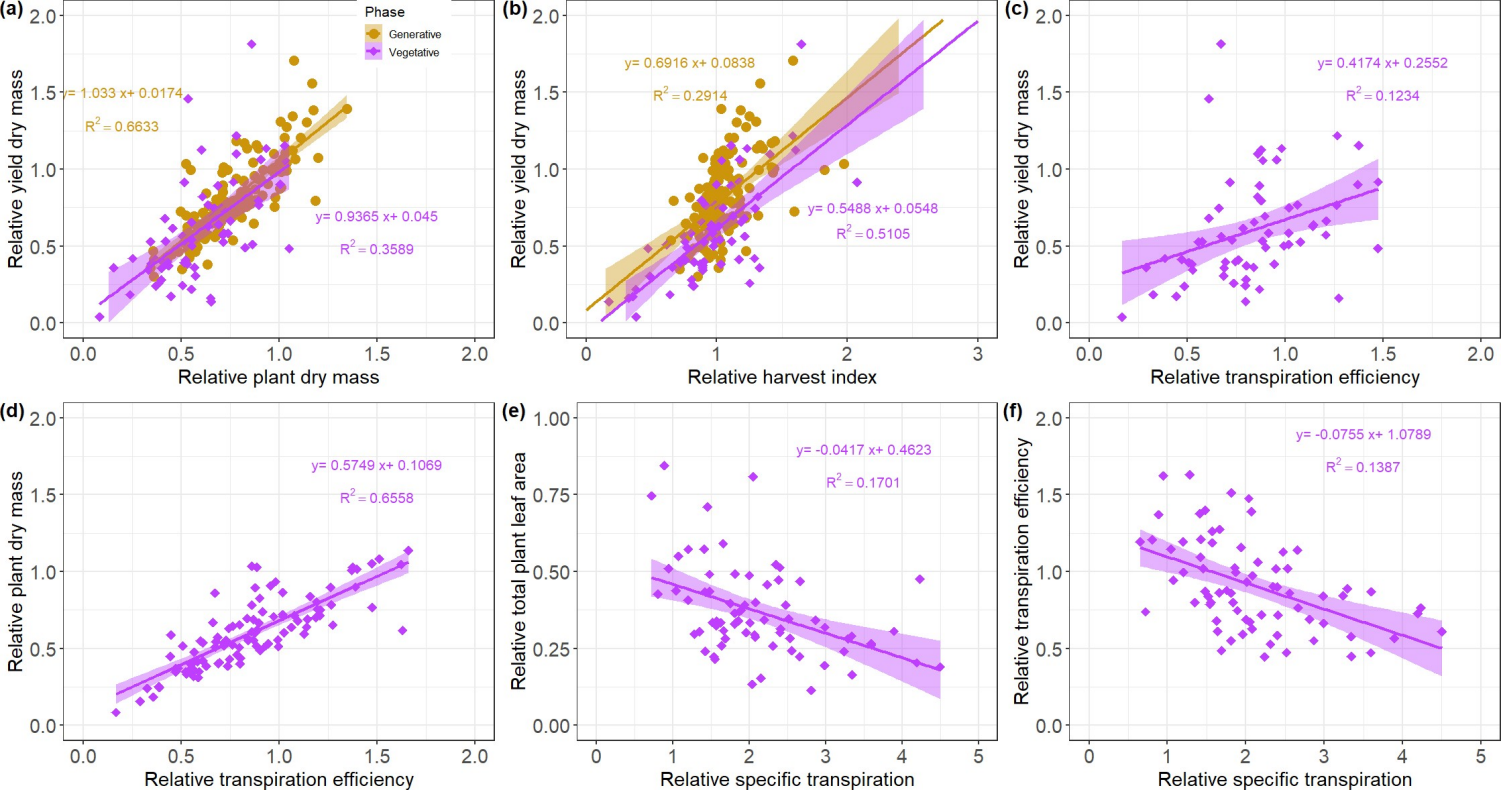
Regression plots between selected relative phenotypic traits for vegetative (violet squares) and generative (yellow bullets) experiments (refer to S4 Fig for the full correlation matrix). (a-c) present the correlation of the relative plant dry mass, relative harvest index and relative transpiration efficiency and the relative yield dry mass; (d) Relative transpiration efficiency and relative plant dry mass; (e-f) relative specific transpiration with the relative total plant leaf area and relative transpiration efficiency.

The relative yield dry mass significantly correlated with traits such as rHI, rDM, rLA, rNt and rDMLG during both vegetative and generative stage experiments (S4 Fig; Fig 5) and weakly correlated with water related traits such as rTE and rT during the vegetative stage experiments. During the vegetative stage experiments, rDM strongly correlated with water related traits (S4 Fig) and especially with rTE (Fig 5D), suggesting that a decay of TE under water shortage would lead to a decay of DM. A weak negative correlation between OA and rDM was found suggesting a decay of DM of the water-limited plant with an increase of OA. The strongest correlation obtained for OA was with rTE (r = -0.25) in the water-limited condition during the vegetative stage (S4A Fig). A weak positive correlation of OA with rPROL was found during the generative stage experiments. rPROL also correlated rLA, rWSC, OA and rDMLG.

The rST significantly correlated with traits such rTE, rLA (Fig 5E and 5F), rNLG, rNt, rDMLG, rDM, rDMS, rSLA and rDMLS (S4A Fig), meaning that the specific transpiration rate plays a key role on the plant performance under water stress. This observation suggests that the increase of the specific transpiration of water-limited plants might accelerate leaf senescence under water deficit. The relative phenological stage appeared to correlate with rLms during both vegetative and generative stage experiments (S4A Fig). The relative yield appeared to correlate stronger with the yield of the tillers than the one of the main stem, probably because the number of tillers was ≥ 2 for most genotypes.

### QTLs associated with phenotypic traits under stress and well-watered conditions

**Fig 6:**
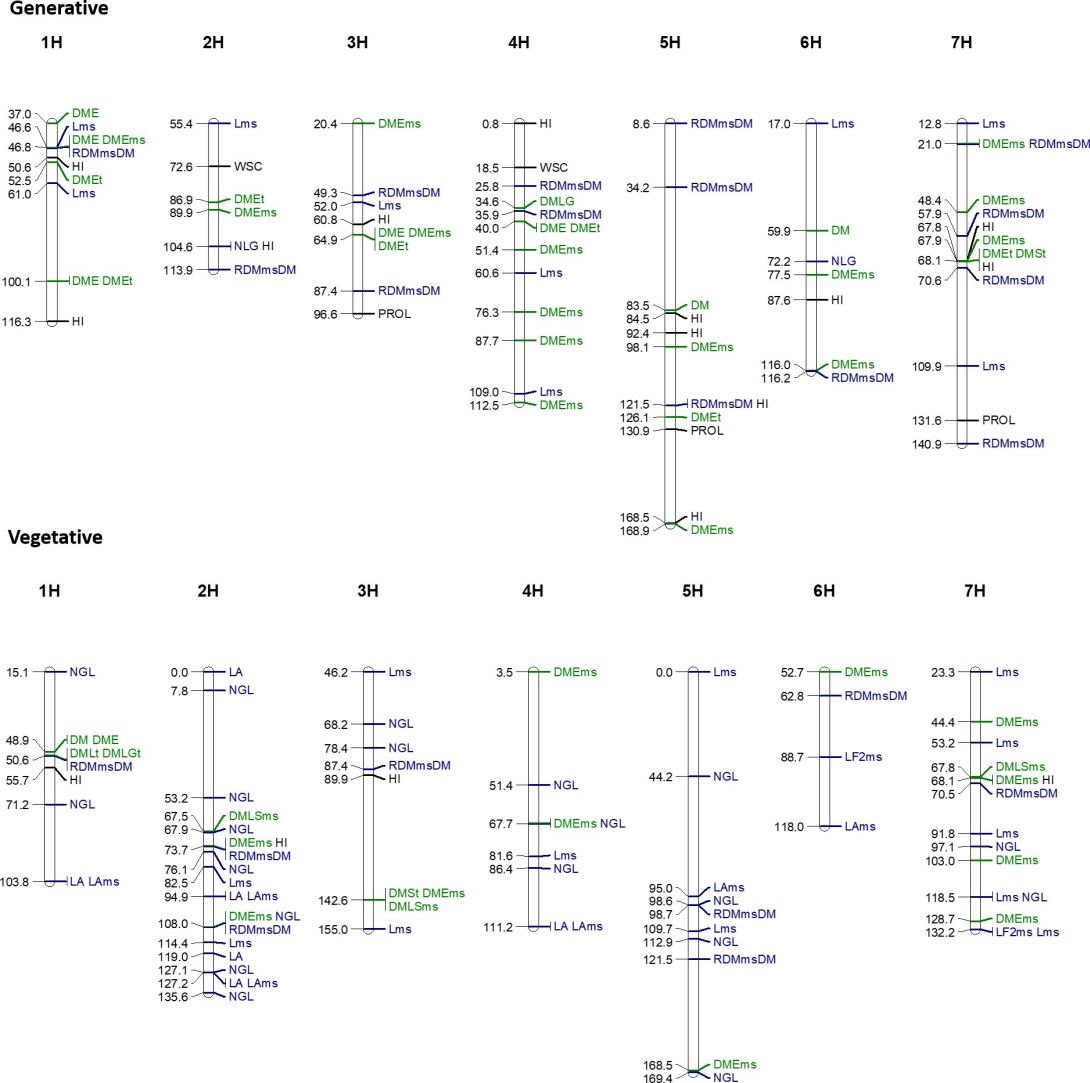
Genome-wide association mapping of phenotype data using a dense genetic map (5892 SNP markers) in both control and water-deficit conditions during the vegetative and generative stage experiments. All QTLs presented here were significant in both stress and control conditions (refer to Tables 3 and 4 for more details about the SNPs). Refers to Table 1 for the acronyms.

GWAS were conducted on all traits showing significant genotypic variation for both well-watered and water deficit treatments. After the vegetative stage treatment, 77 loci associated with 15 phenotypic traits including LA, NLG, DMLGt, LAms and HI were found for harvest data (Fig 6; Table 3).

**Table 3:** Significant marker-trait associations detected under well-watered and drought stress conditions in the vegetative stage experiments for barley genotypes.

Trait	Marker	Chr	Pos	LOD	FDR	Expl. Gen. Variance	Allele 1	Allele 2	WW Allele 1	WW Allele 2	DS Allele 1	DS Allele 2	h^2^	h^2^_se_
**DM**	BOPA1_3689–1101	1H	48.94	7.73	2.72E-05	54.03	0.51	0.87	0.67	0.96	0.34	0.77	0.00	0.00
	Sum					54.03								
**DMLt**	SCRI_RS_198546	1H	50.57	13.01	3.41E-10	72.37	0.18	0.28	0.22	0.41	0.14	0.14	0.00	0.00
	Sum					72.37								
**DMSt**	BOPA1_7803–483	3H	142.63	7.19	6.55E-05	45.46	0.73	1.03	0.98	1.45	0.48	0.61	0.00	0.00
	Sum					45.46								
**DME**	BOPA1_3689–1101	1H	48.94	7.73	2.72E-05	54.03	0.51	0.87	0.67	0.96	0.34	0.77	0.00	0.00
	Sum					54.03								
**DMLGt**	SCRI_RS_198546	1H	50.57	13.01	3.41E-10	72.37	0.18	0.28	0.22	0.41	0.14	0.14	0.00	0.00
	Sum					72.37								
**DMEms**	SCRI_RS_17898	2H	73.73	50.43	2.98E-48	73.81	0.07	0.41	0.12	0.46	0.02	0.37	0.53	0.04
	SCRI_RS_15537	2H	108.00	48.50	1.29E-46	68.84	0.05	0.39	0.10	0.43	0.00	0.34		
	BOPA1_7803–483	3H	142.63	51.97	3.07E-49	77.24	0.43	0.09	0.48	0.14	0.39	0.04		
	BOPA2_12_20274	4H	3.47	9.10	3.32E-09	24.59	0.52	0.21	0.62	0.26	0.42	0.17		
	SCRI_RS_106799	4H	67.71	25.51	8.96E-25	44.80	0.39	0.12	0.44	0.16	0.33	0.08		
	SCRI_RS_120501	5H	168.54	24.75	4.72E-24	44.49	0.43	0.15	0.48	0.20	0.38	0.10		
	SCRI_RS_131341	6H	52.69	36.38	4.67E-35	59.12	0.05	0.36	0.10	0.41	0.00	0.32		
	SCRI_RS_208770	7H	44.41	33.00	7.44E-32	57.06	0.42	0.12	0.47	0.17	0.37	0.08		
	SCRI_RS_158234	7H	68.06	52.32	2.71E-49	77.92	0.06	0.41	0.11	0.45	0.01	0.36		
	GBS5464	7H	103.05	17.24	6.44E-17	38.34	0.40	0.15	0.44	0.20	0.35	0.10		
	GBS5208	7H	128.68	19.63	3.49E-19	41.98	0.42	0.15	0.47	0.20	0.36	0.11		
	Sum					96.78								
**DMLSms**	GBS6809	2H	67.49	25.5	8.6E-24	72.20	0.12	0.19	0.09	0.16	0.14	0.22	0.60	0.05
	BOPA1_7803–483	3H	142.63	34.2	3.6E-31	78.92	0.21	0.12	0.18	0.10	0.24	0.15		
	SCRI_RS_158126	7H	67.78	33.5	8.4E-31	80.11	0.21	0.12	0.17	0.10	0.24	0.15		
	Sum					91.79								
**LF2ms**	SCRI_RS_175839	6H	88.74	3.22	3.55E-01	36.77	39.44	31.92	39.78	30.00	39.11	33.84	0.29	0.17
	SCRI_RS_6430	7H	132.22	3.41	3.55E-01	42.76	38.10	30.99	37.27	29.35	38.92	32.63		
	Sum					48.90								
**Lms**	SCRI_RS_138463	2H	82.51	5.59	3.01E-03	11.49	58.01	51.99	66.17	58.43	49.84	45.55	0.19	0.06
	SCRI_RS_119513	2H	114.38	4.38	6.96E-03	9.60	49.94	56.18	56.00	63.76	43.88	48.61		
	BOPA2_12_30609	3H	46.18	5.49	3.01E-03	12.33	50.71	56.92	57.97	64.16	43.46	49.69		
	BOPA2_12_30055	3H	155.03	3.83	8.61E-03	7.95	63.10	54.03	72.23	61.10	53.96	46.97		
	GBS4691	4H	81.57	3.29	1.68E-02	5.76	59.26	53.80	66.94	60.96	51.57	46.65		
	SCRI_RS_137053	5H	0.00	3.78	9.06E-03	7.26	58.90	53.34	66.49	60.41	51.30	46.27		
	GBS4257	5H	109.65	4.27	6.96E-03	9.85	51.71	57.17	58.05	65.14	45.37	49.21		
	GBS5879	7H	23.30	5.22	4.24E-03	14.69	46.65	56.12	53.35	63.47	39.95	48.77		
	BOPA2_12_30181	7H	53.19	3.92	8.48E-03	9.32	62.12	53.92	71.42	60.92	52.81	46.92		
	GBS2260	7H	91.78	3.40	1.44E-02	7.13	51.18	56.42	57.02	64.17	45.34	48.68		
	GBS2891	7H	118.48	3.81	8.61E-03	11.56	57.88	52.81	66.26	59.36	49.51	46.27		
	BOPA1_ABC11252-1-2-254	7H	132.22	6.51	1.74E-03	14.34	49.28	56.70	55.21	64.37	43.36	49.03		
	Sum					47.69								
**NLG**	BOPA2_12_30588	1H	15.08	5.84	6.29E-05	12.50	3.75	7.24	4.82	10.27	2.68	4.22	0.25	0.05
	SCRI_RS_181239	1H	71.18	4.37	6.68E-04	9.50	8.58	6.45	12.02	9.11	5.13	3.78		
	SCRI_RS_162694	2H	7.79	4.00	1.24E-03	9.75	8.02	6.28	11.35	8.83	4.69	3.72		
	BOPA2_12_30259	2H	53.19	5.26	1.61E-04	13.16	8.15	6.14	11.20	8.87	5.10	3.41		
	SCRI_RS_9469	2H	67.92	8.21	3.51E-06	21.33	8.31	5.85	11.53	8.40	5.09	3.30		
	SCRI_RS_166540	2H	76.06	8.66	2.65E-06	18.48	5.19	7.84	7.32	11.07	3.07	4.61		
	SCRI_RS_15537	2H	108.00	8.27	3.51E-06	21.68	8.37	5.88	11.58	8.46	5.17	3.30		
	GBS1320	2H	127.05	4.65	4.07E-04	10.85	9.30	6.65	13.18	9.38	5.42	3.92		
	SCRI_RS_156155	2H	135.62	4.87	2.89E-04	13.93	8.31	6.28	11.62	8.92	4.99	3.63		
	BOPA2_12_30677	3H	68.24	9.02	2.65E-06	19.06	5.45	8.04	7.69	11.32	3.21	4.76		
	SCRI_RS_198609	3H	78.36	5.45	1.17E-04	13.16	8.02	6.03	11.32	8.50	4.72	3.56		
	BOPA2_12_20156	4H	51.42	9.88	7.40E-07	21.59	4.51	7.66	5.82	10.97	3.20	4.34		
	SCRI_RS_106799	4H	67.71	3.70	2.08E-03	9.11	6.05	7.68	8.76	10.60	3.34	4.76		
	SCRI_RS_138835	4H	86.37	7.68	7.95E-06	18.23	8.35	5.94	11.61	8.49	5.10	3.39		
	SCRI_RS_138556	5H	44.24	4.16	9.50E-04	12.69	8.30	6.39	11.35	9.15	5.26	3.63		
	SCRI_RS_225268	5H	98.63	6.65	2.02E-05	18.77	5.46	7.74	7.78	10.88	3.14	4.61		
	SCRI_RS_212515	5H	112.92	8.55	2.65E-06	20.50	5.25	7.85	7.21	11.20	3.29	4.49		
	BOPA1_2726–852	5H	169.38	6.99	1.55E-05	15.81	5.27	7.68	7.50	10.80	3.03	4.56		
	GBS2693	7H	97.10	6.61	2.02E-05	19.92	5.24	7.75	7.22	11.06	3.25	4.44		
	GBS2889	7H	118.48	3.84	1.65E-03	7.97	8.57	6.51	12.12	9.17	5.03	3.86		
	Sum					44.97								
**LA**	SCRI_RS_218473	1H	103.82	4.40	2.52E-02	11.16	41.58	60.22	62.69	91.05	20.46	29.38	0.18	0.05
	SCRI_RS_187638	2H	0.00	3.81	6.29E-02	5.11	65.94	48.35	99.44	72.82	32.44	23.89		
	BOPA1_5347–585	2H	94.90	3.67	6.41E-02	7.52	45.33	61.03	68.80	92.33	21.86	29.73		
	SCRI_RS_185506	2H	118.97	5.13	1.05E-02	9.95	73.36	49.33	114.20	73.87	32.52	24.79		
	SCRI_RS_155734	2H	127.20	6.04	5.20E-03	11.81	76.03	48.97	118.29	73.02	33.76	24.92		
	GBS5976	4H	111.23	5.58	7.45E-03	14.61	74.16	48.79	118.05	72.45	30.27	25.14		
	Sum					37.85								
**LAms**	SCRI_RS_218473	1H	103.82	6.89	1.21E-04	19.19	18.68	29.58	26.57	42.04	10.79	17.12	0.19	0.06
	BOPA1_5347–585	2H	94.90	2.81	2.11E-02	5.66	22.39	28.61	32.01	40.53	12.76	16.70		
	SCRI_RS_155734	2H	127.20	10.60	1.43E-07	19.25	39.44	22.94	57.63	32.09	21.25	13.79		
	GBS5976	4H	111.23	7.43	5.25E-05	18.51	36.34	23.11	52.78	32.39	19.90	13.83		
	GBS2037	5H	95.00	5.58	9.38E-04	12.55	38.75	23.95	54.28	34.13	23.22	13.76		
	GBS1267	6H	117.99	4.68	2.18E-03	12.33	20.87	29.15	28.82	41.91	12.92	16.39		
	Sum					40.28								
**HI**	SCRI_RS_182431	1H	55.67	19.96	2.83E-17	69.78	0.31	0.18	0.30	0.16	0.32	0.21	0.38	0.07
	SCRI_RS_17898	2H	73.73	19.66	2.83E-17	68.74	0.18	0.31	0.15	0.29	0.20	0.32		
	SCRI_RS_3125	3H	89.87	13.49	1.55E-12	47.99	0.31	0.19	0.29	0.18	0.32	0.21		
	SCRI_RS_158234	7H	68.06	19.06	5.91E-17	67.70	0.17	0.30	0.15	0.29	0.19	0.31		
	Sum					89.46								
**RDMmsDM**	SCRI_RS_198546	1H	50.57	32.38	8.99E-30	70.63	0.68	0.49	0.65	0.36	0.71	0.61	0.21	0.05
	SCRI_RS_17898	2H	73.73	30.21	1.54E-28	66.26	0.48	0.66	0.35	0.62	0.61	0.70		
	SCRI_RS_15537	2H	108.00	29.17	1.09E-27	62.13	0.47	0.65	0.33	0.60	0.60	0.70		
	BOPA1_3791–1525	3H	87.39	23.65	1.00E-22	50.21	0.67	0.51	0.62	0.39	0.71	0.62		
	BOPA2_12_30619	5H	98.72	23.27	2.26E-22	50.19	0.71	0.53	0.68	0.43	0.73	0.63		
	BOPA2_12_30067	5H	121.46	25.61	1.75E-24	53.28	0.70	0.52	0.67	0.41	0.73	0.63		
	BOPA1_8048–952	6H	62.75	27.62	2.98E-26	58.45	0.69	0.51	0.66	0.40	0.72	0.62		
	SCRI_RS_160279	7H	70.54	32.31	8.99E-30	70.43	0.68	0.49	0.65	0.37	0.72	0.61		
	Sum					92.39								
**RDMsDMr**	BOPA1_7803–483	3H	142.63	5.34	1.40E-06	67.47	0.67	0.55	0.69	0.53	0.66	0.57	0.00	0.00
	Sum					67.47								

Refer to Table 1 for all acronyms and units. Allele represents the average marker population value. WW allele 1 and 2: means of the populations with allele 1 respectively allele 2 present under well-watered condition; DS allele 1 and 2: means of the populations with allele 1 respectively allele 2 present under drought stressed condition. Pos.: position (cM); chr.: Chromosome; H^2^: heritability; H^2^_se_: standard error of the broad sense heritability; LOD: log of odds; FDR: False discovery rate

For LA, NLG and LAms, a large number of QTLs were detected, although the sum of the explained genetic variance was below 45% for each of these traits. For DMLGt only one locus was detected which explained 72.3% of genetic variance. No significant loci were found for absolute values of traits such as TE, PROL, OP_100_ or OA. However, co-localisation was found for traits such as DM and DME, which might explain the correlation between both traits. The length of the main stem appeared to be explained by 12 QTL, although the heritability during the experiment was quite weak (19%). After the generative stage water deficit treatment, QTL regions for DM, DME, DMEt, DMEms, DMSt, DMLG, RDMmsDM, NLG, HI, Lms, WSC and PROL were found (Fig 6 and Table 4). For most of these traits, the sums of explained genetic variances were higher than 75%. A total of 85 significant QTLs were found for 13 phenotypic traits. 4 QTLs were found for physiological traits such as PROL and WSC which explained 44% and 37% of the variance, respectively. No QTL was found for OP_100_ and TE and morphological traits such LA and SLA, although genotypic differences were significant for these traits. Most QTLs found were related to ear dry masses. While the marker BOPA2_12_11454 (chromosome 3H, position 64.87 cM) was found to explain a part of the variation in DME, DMEt and DMEms, the marker SCRI_RS_182631 (1H, position 46.81 cM) was only found significant for DME and DMEms suggesting that SCRI_RS_182631 might explain the dry weight production on the main stem ears only, under both water-limited and well-watered conditions. In contrast the QTL BOPA2_12_21003 (4H, position 40.01 cM) was found to explain the variation in the ears of tillers. Significant QTLs for traits such as DMEt were also found to explain the number of tillers Nt, confirming the correlation between both phenotypic traits. However, other markers, which were significant for Nt, were not involved in the grain yield of tillers, although it was significant for DMEms. A total of 9 loci were found to explain the variation of the length of the main stem during both stress and control treatments for a total explained genetic variance of 76%. For the number of green leaves capturing the effect of senescence under both well-watered and stress conditions, 2 significant QTL were found explaining in total 48.3% of the variance.

**Table 4:** Significant marker-trait associations detected under well-watered and drought stress conditions in the generative stage experiments for Barley genotypes.

Trait	Marker	Chr	Pos	LOD	FDR	Expl. Gen. Variance	Allele 1	Allele 2	WW Allele 1	WW Allele 2	WS Allele 1	WS Allele 2	H^2^	H^2^_se_
**DME**	BOPA1_5346–1587	1H	36.97	3.59	1.63E-03	18.00	2.52	1.90	3.07	2.28	1.98	1.53	0.36	0.09
	SCRI_RS_182631	1H	46.81	7.79	1.51E-06	40.52	2.78	1.81	3.28	2.19	2.27	1.42		
	BOPA2_12_30191	1H	100.07	4.91	4.54E-02	29.55	1.98	2.35	2.33	2.69	1.63	2.01		
	BOPA2_12_11454	3H	64.87	12.17	3.62E-09	60.75	1.32	2.41	1.84	2.82	0.80	2.00		
	BOPA2_12_21003	4H	40.01	9.21	1.99E-07	45.99	1.49	2.45	1.94	2.86	1.03	2.05		
	Sum					85.10								
**DMEms**	SCRI_RS_182631	1H	46.81	8.52	1.99E-07	42.89	1.34	0.63	1.96	0.88	0.73	0.38	0.17	0.10
	BOPA2_12_10689	2H	89.94	8.60	1.73E-07	32.59	0.32	1.04	0.78	1.35	-0.13	0.73		
	GBS6049	3H	20.40	7.72	7.94E-07	45.60	0.32	1.06	0.70	1.39	-0.07	0.73		
	BOPA2_12_11454	3H	64.87	11.65	6.01E-09	55.74	0.28	1.08	0.71	1.40	-0.16	0.77		
	BOPA1_3131–1029	4H	51.42	11.33	8.49E-09	52.16	0.28	1.08	0.74	1.38	-0.18	0.78		
	BOPA2_12_30226	4H	76.27	9.60	5.36E-08	39.03	0.25	1.03	0.72	1.34	-0.22	0.72		
	BOPA1_ABC20090-1-1-275	4H	87.70	9.26	7.06E-08	41.78	0.47	1.15	0.81	1.51	0.13	0.78		
	BOPA2_12_31200	4H	112.54	10.14	2.94E-08	47.69	0.29	1.06	0.66	1.40	-0.08	0.72		
	SCRI_RS_157897	5H	98.13	9.29	7.06E-08	42.07	1.16	0.50	1.65	0.70	0.66	0.29		
	SCRI_RS_1928	5H	168.89	11.83	6.01E-09	50.69	1.42	0.61	2.11	0.83	0.72	0.39		
	GBS5031	6H	77.54	9.09	8.28E-08	50.45	1.55	0.70	2.40	0.91	0.69	0.49		
	SCRI_RS_177168	6H	116.01	10.18	2.94E-08	42.24	1.18	0.49	1.66	0.72	0.70	0.26		
	SCRI_RS_179528	7H	20.96	9.07	8.28E-08	52.74	1.26	0.59	1.90	0.79	0.63	0.40		
	SCRI_RS_172335	7H	48.44	8.90	1.05E-07	45.85	0.45	1.11	0.72	1.52	0.18	0.71		
	GBS6627	7H	67.92	10.69	2.24E-08	55.47	1.61	0.68	2.51	0.89	0.70	0.48		
	Sum					103.29								
**DMEt**	BOPA2_12_30191	1H	100.07	4.91	4.54E-02	29.55	1.98	2.35	2.33	2.69	1.63	2.01	0.01	0.13
	SCRI_RS_56976	1H	52.48	6.67	6.92E-05	48.53	1.14	1.68	1.43	2.00	0.86	1.36		
	SCRI_RS_160616	2H	86.86	6.61	6.92E-05	48.01	1.71	1.16	2.05	1.44	1.36	0.87		
	BOPA2_12_11454	3H	64.87	8.09	3.47E-05	61.40	0.81	1.49	1.14	1.81	0.49	1.17		
	BOPA2_12_21003	4H	40.01	6.71	6.92E-05	48.26	0.97	1.51	1.26	1.82	0.69	1.21		
	BOPA2_12_30930	5H	126.13	6.84	6.92E-05	50.87	1.62	1.10	1.98	1.36	1.26	0.83		
	Sum					90.09								
**DM**	BOPA2_12_20403	5H	83.47	5.12	1.50E-02	21.82	4.94	5.99	5.86	6.84	4.02	5.14	0.00	0.00
	BOPA2_12_11386	6H	59.92	5.01	3.84E-02	15.65	4.84	5.75	5.90	6.60	3.78	4.89		
	Sum					21.82								
**DMEt**	SCRI_RS_158234	7H	68.06	14.75	9.74E-12	61.83	1.67	1.19	1.94	1.40	1.41	0.99	0.00	0.00
	Sum					61.83								
**DMSt**	SCRI_RS_158234	7H	68.06	12.41	2.15E-09	48.17	1.95	1.55	2.30	1.91	1.60	1.19	0.00	0.00
	Sum					48.17								
**DMLG**	BOPA2_12_11300	4H	34.56	4.61	1.32E-01	23.53	12.78	5.27	21.68	9.95	3.87	0.59	0.08	0.10
	Sum					23.53								
**RDMmsDM**	SCRI_RS_182631	1H	46.81	11.53	2.46E-09	49.38	0.18	0.45	0.35	0.43	0.00	0.46	0.22	0.11
	BOPA1_9701–925	2H	113.88	11.63	2.46E-09	42.44	0.20	0.46	0.36	0.43	0.04	0.48		
	GBS5497	3H	49.29	8.74	2.80E-07	46.16	0.26	0.49	0.37	0.43	0.14	0.56		
	BOPA1_3791–1525	3H	87.39	7.81	1.46E-06	37.69	0.27	0.48	0.38	0.43	0.16	0.54		
	GBS5051	4H	25.85	5.48	9.11E-05	34.44	0.31	0.52	0.39	0.42	0.22	0.62		
	SCRI_RS_209362	4H	35.91	5.05	1.85E-04	29.00	0.57	0.33	0.43	0.40	0.70	0.26		
	SCRI_RS_169138	5H	8.61	3.41	3.40E-03	23.75	0.49	0.33	0.43	0.40	0.55	0.26		
	GBS6178	5H	34.24	5.30	1.20E-04	32.24	0.54	0.31	0.42	0.39	0.66	0.23		
	BOPA2_12_30067	5H	121.46	12.21	1.65E-09	56.54	0.24	0.48	0.37	0.43	0.11	0.53		
	SCRI_RS_205578	6H	116.15	12.62	1.29E-09	55.23	0.21	0.47	0.36	0.43	0.07	0.51		
	SCRI_RS_179528	7H	20.96	11.50	2.46E-09	50.15	0.20	0.46	0.36	0.43	0.05	0.49		
	SCRI_RS_213842	7H	57.93	11.83	2.46E-09	50.43	0.23	0.47	0.36	0.43	0.09	0.52		
	GBS4229	7H	70.61	11.28	3.19E-09	56.05	0.23	0.49	0.36	0.43	0.09	0.54		
	BOPA1_1847–1745	7H	140.86	3.57	2.58E-03	18.70	0.33	0.52	0.40	0.41	0.26	0.63		
	Sum					95.83								
**NLG**	SCRI_RS_8671	2H	104.60	4.29	8.12E-02	22.87	3.37	5.86	5.39	9.58	1.34	2.14	0.06	0.11
	SCRI_RS_143317	6H	72.24	4.26	8.12E-02	19.87	8.66	4.03	14.20	6.53	3.11	1.53		
	Sum					48.31								
**HI**	SCRI_RS_198546	1H	50.57	11.34	8.15E-09	62.60	0.62	0.31	0.79	0.25	0.45	0.38	0.02	0.09
	SCRI_RS_127646	1H	116.29	8.98	3.01E-07	56.03	0.23	0.58	0.11	0.75	0.36	0.41		
	SCRI_RS_8671	2H	104.60	6.14	3.29E-05	57.21	0.62	0.42	0.79	0.52	0.46	0.33		
	BOPA1_2372–703	3H	60.84	13.64	1.25E-10	68.79	0.29	0.60	0.18	0.80	0.39	0.40		
	GBS1299	4H	0.78	5.18	1.57E-04	29.72	0.62	0.42	0.75	0.53	0.50	0.31		
	GBS3110	5H	84.50	4.97	2.28E-04	31.77	0.73	0.48	0.85	0.59	0.62	0.36		
	GBS3671	5H	92.36	5.00	2.20E-04	31.77	0.73	0.48	0.85	0.60	0.62	0.37		
	BOPA2_12_30067	5H	121.46	5.61	8.08E-05	47.22	0.63	0.44	0.75	0.59	0.52	0.28		
	SCRI_RS_11024	5H	168.54	6.55	1.61E-05	49.69	0.25	0.57	0.12	0.73	0.39	0.40		
	GBS4297	6H	87.61	5.20	1.55E-04	27.54	0.62	0.42	0.74	0.52	0.50	0.31		
	SCRI_RS_158126	7H	67.78	10.20	3.30E-08	62.73	0.59	0.28	0.78	0.17	0.40	0.38		
	SCRI_RS_158234	7H	68.06	12.95	3.01E-10	82.09	0.43	0.37	0.44	0.36	0.41	0.38		
	Sum					94.89								
**Lms**	GBS1779	1H	46.60	6.77	4.56E-04	37.95	66.38	55.57	66.78	56.87	65.99	54.26	0.30	0.09
	BOPA1_11603–445	1H	61.00	4.34	9.82E-03	31.71	54.42	63.81	55.85	64.32	52.99	63.29		
	BOPA1_8889–842	2H	55.38	4.71	6.99E-03	33.20	52.10	63.38	51.64	64.39	52.55	62.37		
	GBS7407	3H	52.03	5.70	2.43E-03	25.23	69.61	57.88	70.76	58.47	68.45	57.29		
	SCRI_RS_206361	4H	60.55	3.67	2.37E-02	15.13	58.15	66.18	58.73	67.15	57.58	65.20		
	GBS5709	4H	109.04	4.41	9.52E-03	24.73	54.04	63.93	55.25	64.34	52.84	63.52		
	BOPA1_1769–545	6H	17.01	3.15	4.01E-02	18.15	57.77	64.91	58.49	65.77	57.05	64.06		
	SCRI_RS_111979	7H	12.75	4.56	8.20E-03	24.08	65.73	56.70	65.93	57.45	65.53	55.95		
	GBS4748	7H	109.92	7.12	4.15E-04	44.95	65.67	54.60	66.95	54.53	64.38	54.67		
	Sum					76.11								
**PROL**	SCRI_RS_172266	3H	96.60	4.07	2.23E-02	15.66	33.71	20.06	2.11	1.67	65.31	38.46	0.01	0.10
	SCRI_RS_161534	5H	130.90	3.97	2.57E-02	13.47	34.53	20.24	1.76	1.80	67.30	38.69		
	GBS4530	7H	131.59	5.79	9.09E-03	21.10	44.08	20.85	1.57	1.83	86.59	39.87		
	Sum					43.92								
**WSC**	SCRI_RS_7517	2H	72.59	3.92	2.15E-01	19.11	304.74	219.99	329.10	236.96	280.38	203.01	0.15	0.12
	SCRI_RS_195137	4H	18.48	4.91	6.68E-02	24.01	302.77	217.51	312.39	236.32	293.16	198.71		
	Sum					37.12								

Refer to Table 1 for all acronyms and units. Allele represents the average marker population value. WW allele 1 and 2: means of the populations with allele 1 respectively allele 2 present under well-watered condition; DS allele 1 and 2: means of the populations with allele 1 respectively allele 2 present under drought stressed condition. Pos.: position (cM); chr.: Chromosome; H^2^: heritability; H^2^_se_: standard error of the broad sense heritability; LOD: log of odds; FDR: False discovery rate

### Heritability

Environmental variation across water supply treatments were investigated for each trait. The broad sense heritability (H^2^) revealed large variation across the treatments and traits classes, ranging from 0.0 to 0.60 in the vegetative experiment, from 0.0 to 0.38 during the generative experiment, and from 0.0 to 0.99 across the treatments (Tables 3–5). H^2^ of relative trait was relatively higher, ranging from 0.0 to 0.99 compared to that of trait values. The heritability of the traits without significant QTLs was not investigated. The relative proline content had the highest heritability although the proline content had a very low heritability (0.01) during the generative experiment. H^2^ was also experimental stage dependent, varying between the generative and the vegetative experiment for each investigated trait.

**Table 5:** Significant marker-trait associations for relative values explaining the phenotypic plasticity of different physiological and morphological traits of barley genotypes after water deficit treatment during the generative growth stage.

Trait	Marker	Chr	Pos	LOD	FDR	Expl. Gen. Variance	Allele 1	Allele 2	H^2^	H^2^_se_
**rPROL**	GBS6990	2H	40.66	4.23	4.19E-02	9.36	162.60	69.43	0.99	0.00
	GBS2303	2H	51.20	6.35	1.25E-03	15.80	224.27	70.29		
	BOPA2_12_30370	3H	139.67	4.41	3.13E-02	10.03	193.47	73.67		
	GBS2307	6H	87.61	5.04	9.22E-03	12.90	153.32	65.79		
	GBS785	7H	13.88	6.75	1.02E-03	16.71	172.44	65.41		
	**Sum**					32.01				
**OA**	SCRI_RS_15537	2H	108.00	5.08	1.76E-02	63.53	0.18	-0.07	0.03	0.11
	BOPA1_6951–875	2H	90.26	20.12	3.53E-19	39.96	-0.27	-0.01		
	SCRI_RS_144379	2H	111.26	31.04	5.20E-29	55.39	-0.24	0.03		
	BOPA1_6954–861	4H	52.96	30.84	7.50E-29	55.09	-0.28	0.01		
	SCRI_RS_188829	4H	115.23	21.43	2.08E-20	44.94	0.06	-0.16		
	BOPA1_8048–952	6H	62.75	34.78	9.43E-32	59.72	-0.24	0.03		
	BOPA1_2924–1189	7H	70.68	32.94	3.06E-30	58.44	-0.22	0.04		
	**Sum**					85.66				
**rWSC**	GBS3887	2H	58.05	6.61	6.02E-04	14.62	1.45	1.05	0.14	0.09
	BOPA1_2822–739	2H	123.94	7.28	2.95E-04	9.64	1.16	0.79		
	BOPA2_12_30895	7H	34.35	4.90	4.99E-03	10.13	0.66	0.96		
	**Sum**					23.86				
**rBBCH**	SCRI_RS_158234	7H	68.06	6.09	6.91E-04	72.23	0.97	1.04	0.00	0.00
	**Sum**					72.23				
**rDME**	SCRI_RS_183064	2H	110.91	14.71	3.53E-12	65.90	0.99	0.70	0.00	0.00
	BOPA1_ABC03113-1-1-251	5H	129.44	14.83	3.53E-12	66.46	1.04	0.72		
	**Sum**					80.84				
**rDMEms**	BOPA2_12_30191	1H	100.07	36.95	6.28E-34	71.57	0.60	1.21	0.08	0.11
	SCRI_RS_185710	2H	58.07	26.16	2.75E-24	57.46	0.77	1.24		
	SCRI_RS_3125	3H	89.87	27.50	1.96E-25	58.94	0.80	1.26		
	BOPA2_12_30158	4H	99.08	21.60	3.65E-20	54.44	0.78	1.23		
	BOPA1_4773–1009	4H	112.33	28.82	4.18E-26	58.31	0.64	1.20		
	SCRI_RS_167426	5H	143.96	6.78	1.00E-06	17.72	0.90	1.17		
	BOPA1_2607–2929	6H	79.60	12.04	1.85E-11	30.80	1.25	0.93		
	GBS5694	7H	69.26	28.35	7.00E-26	63.51	0.72	1.23		
	Sum					93.51				
**rDMGLt**	BOPA2_12_20489	2H	72.45	8.24	3.04E-05	44.53	2.09	0.36	0.18	0.15
	**Sum**					44.53				
**rDMLSms**	BOPA2_12_30191	1H	100.07	39.00	5.56E-36	74.90	2.06	1.10	0.17	0.10
	GBS6733	2H	58.78	26.11	5.41E-24	62.34	1.77	1.05		
	SCRI_RS_143727	2H	107.15	15.99	7.00E-15	45.15	1.74	1.11		
	GBS6415	4H	40.01	28.93	2.16E-26	64.27	2.02	1.11		
	SCRI_RS_179438	4H	73.65	7.02	8.60E-07	13.66	1.10	1.49		
	SCRI_RS_209980	5H	44.24	8.88	1.82E-08	18.80	1.58	1.13		
	SCRI_RS_143508	5H	93.65	11.36	9.67E-11	29.58	0.99	1.49		
	SCRI_RS_198617	5H	96.25	5.85	9.07E-06	12.86	1.61	1.19		
	BOPA2_12_11386	6H	59.92	21.97	2.00E-20	49.09	2.18	1.18		
	GBS4671	6H	117.55	15.57	1.65E-14	43.26	1.71	1.10		
	GBS5694	7H	69.26	28.58	3.63E-26	63.00	1.85	1.07		
	BOPA1_3140–491	7H	77.97	4.16	2.73E-04	10.62	0.93	1.36		
	**Sum**					96.93				
**rNLG**	BOPA2_12_10717	2H	65.37	24.23	3.12E-21	83.75	6.00	0.42	0.38	0.11
	**Sum**					83.75				
**rRDMEmsDME**	GBS6407	1H	54.89	32.22	1.44E-30	68.92	1.11	1.71	0.12	0.11
	BOPA1_3598–489	2H	50.92	28.22	7.80E-27	61.22	1.79	1.21		
	SCRI_RS_237688	2H	57.17	18.97	3.31E-18	44.84	1.01	1.58		
	SCRI_RS_17898	2H	73.73	40.94	1.59E-38	74.49	1.73	1.10		
	SCRI_RS_17898	2H	73.73	40.94	1.59E-38	74.49	1.73	1.10		
	BOPA1_ConsensusGBS0508-1	3H	51.35	21.90	6.05E-21	50.47	1.04	1.60		
	GBS881	6H	28.47	24.90	1.01E-23	57.47	1.11	1.67		
	SCRI_RS_158126	7H	67.78	42.67	3.99E-40	75.94	1.09	1.73		
	SCRI_RS_158234	7H	68.06	45.63	1.32E-42	78.90	1.76	1.12		
	**Sum**					95.88				
**rRDMmsDM**	BOPA2_12_30191	1H	100.07	31.30	2.81E-28	66.81	1.20	1.29	0.00	0.12
	SCRI_RS_185710	2H	58.07	24.24	1.62E-22	55.04	1.22	1.30		
	GBS2300	2H	80.95	20.69	2.43E-19	52.61	1.20	1.29		
	SCRI_RS_3125	3H	89.87	28.76	4.86E-26	61.50	1.23	1.30		
	SCRI_RS_135425	5H	45.21	13.61	6.08E-13	35.93	1.31	1.25		
	GBS6627	7H	67.92	14.19	1.76E-13	38.82	1.20	1.28		
	GBS5694	7H	69.26	27.16	9.78E-25	62.90	1.21	1.29		
	BOPA2_12_31513	7H	70.96	23.65	5.44E-22	55.50	1.22	1.29		
	SCRI_RS_204144	7H	70.96	15.60	1.01E-14	40.75	1.24	1.30		
	**Sum**					95.90				
**rDMSt**	BOPA2_12_30191	1H	100.07	35.67	1.20E-32	71.11	0.63	0.80	0.01	0.12
	SCRI_RS_185710	2H	58.07	26.15	2.05E-24	58.42	0.68	0.81		
	GBS2300	2H	80.95	20.34	4.55E-19	50.53	0.64	0.80		
	SCRI_RS_3125	3H	89.87	28.91	1.73E-26	61.75	0.69	0.82		
	SCRI_RS_232881	5H	45.21	12.59	5.51E-12	33.62	0.82	0.73		
	BOPA2_12_30857	6H	56.16	9.14	7.06E-09	24.41	0.81	0.73		
	GBS5694	7H	69.26	29.56	7.74E-27	65.05	0.66	0.81		
	BOPA2_12_31513	7H	70.96	22.16	1.14E-20	49.59	0.67	0.80		
	SCRI_RS_204144	7H	70.96	13.67	5.45E-13	36.40	0.71	0.81		
	**Sum**					96.09				
**rNt**	BOPA2_12_30191	1H	100.07	29.51	2.43E-27	63.09	0.93	1.07	0.00	0.12
	SCRI_RS_237688	2H	57.17	26.25	1.58E-24	58.13	0.95	1.07		
	SCRI_RS_9469	2H	67.92	29.94	1.59E-27	62.18	1.10	0.99		
	SCRI_RS_3125	3H	89.87	29.65	2.09E-27	61.96	0.97	1.08		
	SCRI_RS_158234	7H	68.06	32.47	1.90E-29	65.29	1.09	0.98		
	Sum					92.74				
**SLAt**	SCRI_RS_196910	1H	117.49	5.14	2.02E-02	14.62	129.62	157.98	0.00	0.00
	SCRI_RS_171038	2H	80.59	3.50	4.21E-02	10.76	135.44	157.53		
	SCRI_RS_106163	2H	129.75	3.85	4.17E-02	10.26	163.47	139.19		
	SCRI_RS_137464	6H	55.67	3.47	4.25E-02	8.92	134.37	156.79		
	BOPA1_6541–1329	7H	100.00	5.26	2.02E-02	14.59	172.29	140.09		
	**Sum**					40.95				

Allele represents the average marker population value. Refer to Table 1 for all acronyms and units. Allele 1 and 2: means of the populations with allele 1 respectively allele 2. Pos.: position (cM); chr.: Chromosome; H^2^: heritability; H^2^_se_: standard error of the broad sense heritability; LOD: log of odds; FDR: False discovery rate

### QTLs associated with the response to water stress

**Fig 7:**
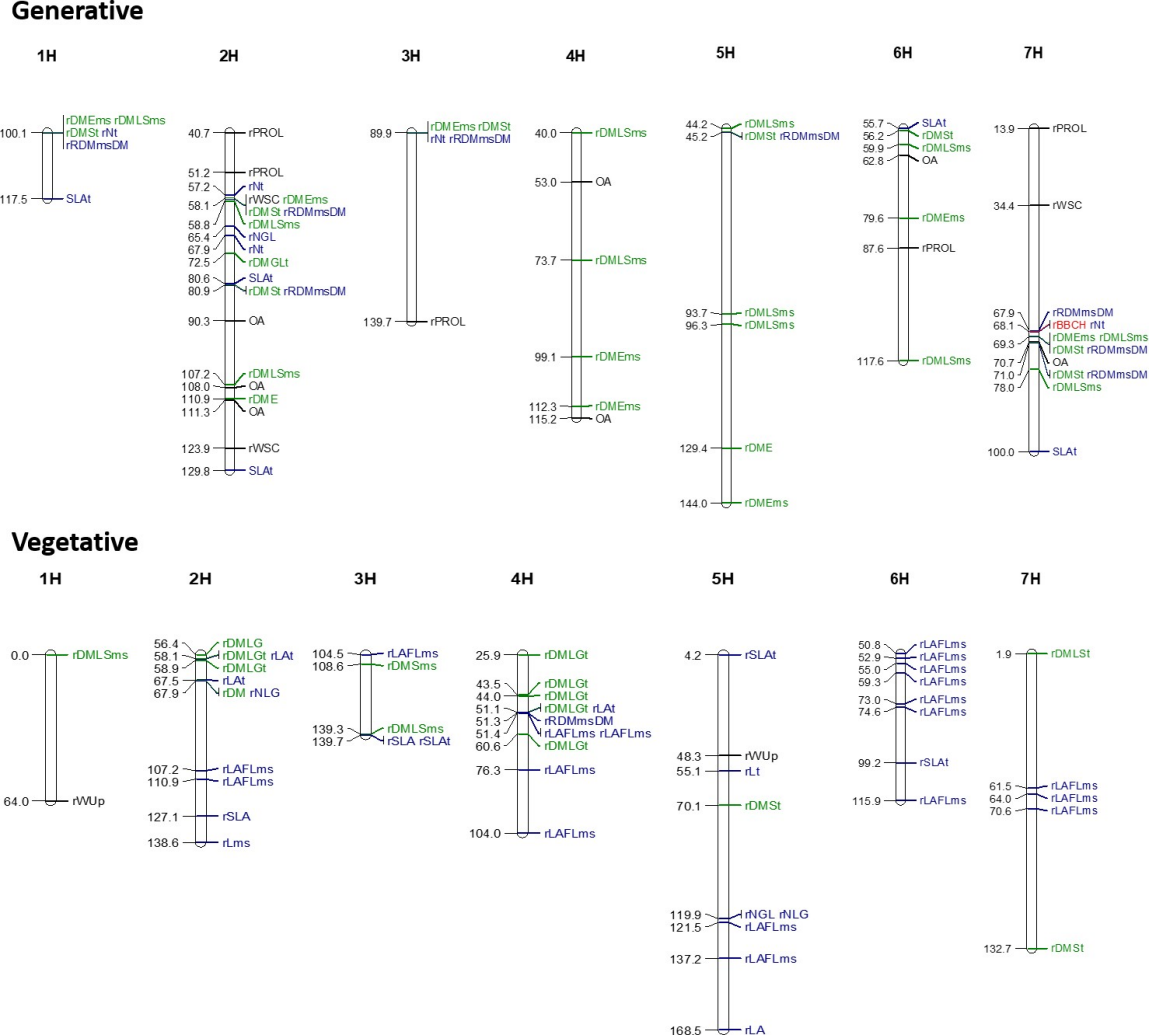
Genome-wide association mapping of relative phenotype data presenting significant GxT interaction using a dense genetic map (5892 SNP markers, refers to Table 5 and 6 for more details about the SNPs and to Table 1 for the acronyms) during both the generative and the vegetative stage experiments.

GWAS were also performed on relative values to assess genotypic responses under water shortage, for traits with a significant GxT interaction and a significant genotypic difference on relative values after the control and stress treatments in the generative and vegetative stage experiments. After the generative stage experiment, 58 significant loci were found for 14 traits including functional and physiological traits (Fig 7 and Table 5 for more details). Some markers such as BOPA2_12_30191, BOPA2_12_31513, GBS2300, GBS5694, SCRI_RS_158234, SCRI_RS_185710, SCRI_RS_237688, SCRI_RS_3125, SCRI_RS_204144, SCRI_RS_185710 and SCRI_RS_17898 were significant for more than one trait. One single locus (SCRI_RS_158234) located on chromosome 7 was found to be significant for the relative phenological stage suggesting that the QTL is leading for example to an accelerated senescence or drought escape strategy under drought stress. Fifteen significant loci for stress indexes of physiological traits such as OA, rPROL and rWSC were found. After stress treatments in the vegetative stage experiments, 55 QTLs were significant (LOD>3.0) for 17 phenotypic traits. Thirteen markers were repeated for several phenotypic traits (Fig 7 and Table 6 for more details).

**Table 6:** Significant marker-trait associations for relative values explaining the phenotypic plasticity of different physiological and morphological traits of barley genotypes after water deficit treatment during the vegetative growth stage.

Trait	Marker	Chr	Pos	LOD	FDR	Expl. Gen. Variance	Allele 1	Allele 2	H^2^	H^2^_se_
**rDM**	SCRI_RS_9469	**2H**	67.92	5.69	2.14E-03	74.95	0.55	0.66	0.00	0.00
	Sum	** **				74.95				
**rDMGLt**	BOPA1_4665–882	**1H**	67.92	13.16	4.64E-11	70.57	1.53	0.18	0.83	0.05
	BOPA2_12_30191	**1H**	100.07	13.65	4.64E-11	71.41	1.58	0.20		
	GBS7416	**2H**	56.73	13.23	4.64E-11	69.71	1.61	0.26		
	GBS6809	**2H**	67.49	13.00	5.86E-11	70.16	-0.02	1.23		
	Sum	** **				92.43				
**rLAFL1ms**	BOPA2_12_31034	**5H**	48.33	7.26	9.08E-05	43.25	1.57	1.01	0.99	0.00
	Sum	** **				43.25				
**RLALGt**	GBS1230	**2H**	58.07	13.08	1.30E-10	68.54	1.14	0.14	0.78	0.07
	GBS6809	**2H**	67.49	12.18	1.94E-10	69.10	-0.06	0.84		
	GBS5733	**4H**	51.13	13.14	1.30E-10	67.71	1.31	0.18		
	Sum	** **				94.70				
**rDMLG**	SCRI_RS_219740	**2H**	56.37	4.85	5.97E-03	65.36	0.69	0.47	0.00	0.00
	Sum	** **				65.36				
**rRDMmsDM**	GBS508	**4H**	51.27	7.64	3.42E-05	76.30	1.19	1.69	0.00	0.00
	Sum	** **				76.30				
**rLA**	SCRI_RS_120501	**5H**	168.54	3.76	1.45E-02	52.64	0.44	0.33	0.00	0.00
	Sum					52.64				
**rNLG**	SCRI_RS_9469	**2H**	67.92	4.44	2.42E-02	72.96	0.41	0.57	0.04	0.14
	GBS4361	5H	119.93	5.94	6.29E-03	23.70	0.64	0.41		
	Sum	** **				72.96				
**rDMGLt**	GBS1230	2H	58.07	21.13	4.05E-18	68.64	1.11	0.33	0.21	0.12
	GBS2119	2H	58.92	17.38	1.42E-15	60.72	0.97	0.31		
	GBS5051	4H	25.85	12.45	2.31E-11	50.91	0.82	0.28		
	GBS5473	4H	43.48	13.08	7.71E-12	50.22	0.88	0.30		
	GBS3009	4H	44.00	20.46	4.74E-18	67.29	1.06	0.32		
	GBS5733	4H	51.13	20.71	4.74E-18	68.18	1.29	0.37		
	SCRI_RS_239145	4H	60.55	4.72	1.17E-04	33.49	0.23	0.70		
	Sum					92.37				
**rDMLSms**	BOPA2_12_30968	1H	0.00	5.36	2.41E-02	20.00	8.34	2.09	0.89	0.02
	BOPA2_12_20505	3H	139.31	4.77	4.69E-02	17.77	6.91	2.05		
	Sum					32.51				
**rDMLSt**	GBS2306	7H	1.91	5.42	2.10E-02	23.30	15.49	2.46	0.96	0.01
	Sum					23.30				
**rDMSms**	SCRI_RS_165334	3H	108.57	6.35	2.45E-03	23.42	1.01	0.71	0.07	0.13
	Sum					23.42				
**rDMSt**	GBS4766	5H	70.14	4.96	6.69E-03	2.85	6.98	0.54	0.92	0.01
	GBS1121	7H	132.65	4.50	1.45E-02	18.91	1.50	0.53		
	Sum					19.25				
**rLAFL2ms**	BOPA2_12_11285	2H	89.80	4.93	3.22E-02	20.47	3.22	0.96	0.58	0.06
	GBS1162	6H	91.05	5.19	3.22E-02	22.84	4.04	0.98		
	Sum					35.48				
**rLAFLms**	BOPA1_2277–634	2H	107.15	13.74	3.28E-11	51.96	1.06	0.91	0.01	0.15
	SCRI_RS_183064	2H	110.91	14.03	2.50E-11	53.87	1.08	0.92		
	BOPA2_12_30926	3H	104.46	9.42	4.37E-08	38.24	1.06	0.93		
	BOPA1_3131–1029	4H	51.42	8.93	1.06E-07	32.68	0.80	0.99		
	BOPA2_12_20156	4H	51.42	14.38	2.26E-11	59.88	1.10	0.93		
	BOPA2_12_30226	4H	76.27	9.38	4.47E-08	34.39	0.78	0.99		
	SCRI_RS_7914	4H	103.97	7.74	8.01E-07	30.71	1.03	0.92		
	BOPA2_12_30067	5H	121.46	12.80	8.01E-11	49.98	1.09	0.93		
	SCRI_RS_166491	5H	137.22	8.30	3.11E-07	31.34	0.85	1.00		
	SCRI_RS_161288	6H	50.78	3.63	1.46E-03	14.94	1.03	0.94		
	SCRI_RS_196373	6H	52.90	3.53	1.76E-03	15.11	1.03	0.94		
	BOPA1_6487–1315	6H	55.03	7.67	9.31E-07	30.41	0.90	1.01		
	BOPA1_1914–936	6H	59.26	12.56	1.11E-10	48.51	1.05	0.91		
	SCRI_RS_81903	6H	72.95	6.87	3.83E-06	26.31	0.91	1.02		
	SCRI_RS_206976	6H	74.58	7.98	5.34E-07	32.77	1.05	0.93		
	BOPA1_1007–651	6H	115.93	4.56	2.65E-04	20.48	1.04	0.94		
	SCRI_RS_116905	7H	61.47	13.52	4.09E-11	53.24	1.09	0.93		
	BOPA1_ABC14535-1-1-75	7H	63.95	8.05	4.80E-07	33.58	1.07	0.94		
	GBS4229	7H	70.61	11.11	1.68E-09	46.78	1.09	0.93		
	Sum					98.85				
**rLms**	GBS5720	2H	138.60	6.29	2.85E-03	25.30	1.39	0.76	0.16	0.12
	Sum					25.30				
**rLt**	GBS3514	5H	55.10	7.12	4.13E-04	28.98	1.28	0.60	0.07	0.13
	Sum					28.98				
**rNLG**	GBS4361	5H	119.93	5.94	6.29E-03	23.70	0.64	0.41	0.04	0.14
	Sum					23.70				
**rSLA**	GBS1319	2H	127.05	4.82	4.19E-02	19.65	1.06	0.69	0.06	0.14
	BOPA2_12_30370	3H	139.67	9.65	1.23E-06	38.45	1.35	0.69		
	Sum					46.14				
**rSLAt**	BOPA2_12_30370	3H	139.67	5.85	3.81E-03	30.81	2.11	0.67	0.28	0.11
	BOPA2_12_31326	5H	4.17	7.05	4.85E-04	29.02	2.24	0.67		
	GBS7027	6H	99.15	4.91	1.35E-02	21.59	2.03	0.66		
	Sum					48.08				
**rT**	GBS2202	1H	64.02	5.97	5.83E-03	24.31	1.32	0.74	0.06	0.13
	GBS4670	5H	48.26	5.32	1.32E-02	21.14	1.29	0.73		
	Sum					37.45				

Refer to Table 1 for all acronyms and units. Allele represents the average marker population value for each allele. Refer to Table 1 for all acronyms and units. Allele 0 and 1: means of the populations with allele 0 respectively allele 1. Pos.: position (cM); chr.: Chromosome; H^2^: heritability; H^2^_se_: standard error of the broad sense heritability; LOD: log of odds; FDR: False discovery rate

## Discussion

The yield decline under water deficit was widely reported for several crops including barley in the literature. However, the quantification of the physiological and morphological responses of different spring barley genotypes to water deficit as well as the characterization of the genetic base to find QTL for the investigated traits is still not explored. In this work, we aimed at investigating the response of relevant traits under water deficit to identify corresponding QTLs.

### Physiological and morphological responses to water deficit

Water deficit had a significant effect on phenological stages (BBCH scale), morphological and functional traits of barley, especially during the vegetative stage experiments (Table 2). The effect of drought stress on BBCH scale agrees with [23] where a decrease of the grain filling time and an early grain maturity under water shortage were reported, which might be observed through a change in BBCH stage. The decrease of the plant biomass due to drought stress observed in this study was reported in several other studies [30, 50, 81] and the positive correlation between TE and biomass production as well [13]. The strong correlation between rDMmsDM and rDM (S4 Fig) or DMmsDM and DM (S3 Fig) in the generative stage experiments showed that water deficit reduced total plant biomass mainly by reducing tiller biomass. During the vegetative stage experiments, the highly significant genotypic effects on the relative lengths of most leaves including the flag leaf of the main stem (Table 2) might indicate differences in the sensitivity of cell expansion among the genotypes.

Interestingly, there was no significant water deficit effect on HI (Table 2). In fact, water deficit accelerated the flowering in dehydration-avoidant plants as reported in the literature [9], which can lead to differences in ear dry mass of control and stress treatments at harvesting time. During the generative stage experiments, the stress treatment started with spiking. Maybe an earlier stress treatment could have a significantly negative effect on grain filling period of water saving genotypes with an accelerated senescence and WSC translocation.

On the other hand, we found that genotypes with higher dry weight production under drought stress were mostly plants with high transpiration and TE under both stress and control conditions (Fig 4). Several genotypes were found to improve significantly TE under water deficit, although DM was still low. An analysis of the root system of these varieties might help to understand where the dry weight was allocated.

The correlation between rTE and rDM (Fig 3D) showed that increasing the plant biomass production under water shortage was essentially a function of increased TE rather than improved water uptake capacity (represented by the maximum water extraction [%]). Because available water was identical for all genotypes of the stress treatment and the water uptake capacity of the genotypes was in a close range between 70 and 90% of 1.78 litre (excluding some genotypes in the generative stage with a lower total amount of water transpired, which may occur because plants were fully grown earlier), the efficiency of biomass production from the transpired water is a key trait. This is substantiated by the statistical analysis (Table 2), which showed significant genotypic effects for relative TE but not for Wex.

According to Passioura [20], next to the transpiration and TE, HI is the third component explaining grain yield. rHI was reduced in some genotypes and increased in others (Fig 2D). A relative plant dry mass less than 1 in combination with rHI bigger than 1 indicated, that grain filling was less affected by the drought stress than growth of other plant organs. Drought tolerant cultivars should be the varieties having less reduction in grain yield and related traits. Therefore, variation of rDME and rHI as observed in Fig 1 might be related to tolerance to water deficit.

An increase in proline under water deficit as observed in this work was frequently reported [11, 50]. The weak negative correlation between rPROL of water-limited plants and rDM found in our study confirmed the observation that proline production under water deficit did not lead to a significantly higher dry matter production. Therefore, proline production might be associated with several other physiological responses leading to adaptation.

The increase of water-soluble carbohydrates in most genotypes in the water deficit treatment was also reported in the literature [32, 33, 82]. However, only a weak positive correlation between WSC content in stems and proline content in leaves of the water-limited plants was found (r = 0.24). During the water deficit treatment, WSC was translocated from stems to developing grains [65], resulting to a reduction of WSC content in the stems. Yang et al. [83] reported that drought stress during grain filling of wheat led to faster and better remobilization of pre-stored carbon from vegetative tissues to grains. In our experiments, the percentage of WSC translocated from the stem varied from 0 to over 75%. The negative correlation between relative WSC in the stems and absolute ear dry mass of the water-limited plants (S3 Fig) showed that highest DME was obtained when WSC content in stems was reduced during the drought treatment (rWSC < 1), which can be interpreted as an translocation efficiency of WSC from stems to grains. Genotypes with an increased WSC content in stems during the drought stage (rWSC > 1) might accumulate WSC in stems as compatible solutes against drought or may have transport or sink limitations.

No relationship between OA and stability of biomass production under water deficit [30] or other benefits of OA [34, 27] were found in our experiments. Wehner et al. [50] also found no direct relationship between OA and biomass production under water deficit. This might have been due to the high variance of OA between experiments, suggesting that OA is very sensitive to the environment. Moreover, OA might vary also at plant level with leaf age.

The specific transpiration was found to negatively correlating with most leaf level traits such as LA, DMLG, NLG and SLA. However, no relationship with the total amount of water use was found. The relationship between the specific transpiration and the stomatal conductance could allow us to better assess the role of ST under water deficit.

### QTL marker information and heritability

Marker-trait associations on all chromosomes were found for DMEms, showing that this trait is the result of a multitude of processes on all chromosomes, irrespective of water supply. Tondelli et al. [84] found QTL on chromosomes 1H, 2H, 5H and 6H for this trait, Yin et al. [46] found QTL on chromosomes 1H, 2H, 3H, 4H, 5H and 6H, while Mora et al. [85] could identify QTL on chromosome 1H and 3H only. Xue et al. [52] identified QTL for grain yield of control and waterlogged conditions on chromosome 2H (position around 83 cM) only. Marker-trait associations for DMEt and DMEms were also found in this study on chromosome 2H (positions 86.86 and 89.94 cM), but in addition to the results of Xue et al. [52] several marker-trait associations for grain yield were also found on other chromosomes (S1 Table). Comparing the QTL for grain yield from this study with the results of Tondelli et al. [84] was not possible because of missing information about chromosome positions.

The markers explaining the largest proportions of genetic variance for LA were identical with markers for LAms, but no marker-trait associations for leaf area of tillers were found. This underscores the importance of the main stem in regulating leaf expansion. In a study aiming to identify QTLs for physiological and morphological traits of flag leaves at the pre-filling stage [77], QTL for flag leaf area only were found on chromosome 2H (position 77.2 cM). This is not in agreement with the results of QTLs for total LA in our study. No marker-trait association was found for tiller number in our dataset. Other experiments could identify two QTL for tiller number on chromosomes 3H and 4H [53].

Mora et al. [79] found QTL for HI of fully irrigated plants on chromosome 5H and 3H (positions 61, 64 and 67) while the strongest QTL for HI in this study was also found on chromosome 3H (position 60.84 cM) and four marker-trait associations for HI where also found on chromosome 5H. The markers found for grain yield by Mora et al. [85] on chromosome 1H (position 140 cM) could not be verified.

For OA, 5 QTLs were found in our dataset on chromosomes 2H, 4H, 6H and 7H after the generative stage treatment. Some QTLs were reported for this trait [36, 42] on chromosome 3H, 6H and 7H. Certainly, the results are often hard to compare because of the high experimental variation and/or differences in sampling or leaf age or BBCH stage at drought initiation. In some cases results of QTL analysis are shown without marker position (cM), which is another difficulty for comparison [85].

According to different brad sense heritability values, variations of most dry weight and water relation traits were largely due to the environment, and the variation of the relative response of traits were mainly genetic (Tables 3 to 6). The heritability of traits such as rPROL was surprisingly very high (0.99, Table 5) suggesting a strong genetic control depending on the signals of soil water status. High H^2^ of rPROL suggests that average differences between genotypes are large compared with yearly variation within genotypes. This is consistent with the broad range of rPROL observed between genotypes.

## Conclusion

Physiological and morphological response to drought stress on spring barley genotypes were found, including the reduction of relative plant dry mass, decrease of relative leaf area and increase of proline content in the leaves. The positive relationship of TE and plant dry mass production was confirmed in this study. TE had a major impact on dry mass production compared to the total amount of water transpired in these experiments and was reduced by 22% under drought stress. No clear relationship was found for OA, although it was reported to promote stability of biomass production and other benefits under drought stress conditions.

Significant marker-trait associations for a part of the investigated traits involved in drought stress responses such as total leaf area, dry mass of ears (grain yield), harvest index and length of the main stem, water soluble carbohydrate, specific leaf area, and the dry mass of senescent leaves on the main stem were detected. Over 110 new markers associated with the GxT interaction were detected for physiological and morphological traits. To the best of the author knowledge, some of the QTLs found such as those for grain yield (chromosome 4H, position 40) or HI (chromosome 7H) have not yet been reported for these traits in the literature. The identified genomic regions found in this study can be used for marker-assisted selection in barley to improve drought tolerance in the future. The physiological and genetic information from these experiments could also be used to develop genetic based physiological equations for modelling of plant stress responses *in silico*.

## Supporting information

S1 Table Details about initiation of the experiments and harvest dates. (DOCX)

S2 Table Analysis of variance for genotypic and treatment effects and their interactions on different absolute plant traits using a linear mixed model during the both the generative and vegetative stage experiments. (DOCX)

S3 Table Analysis of variance (ANOVA) of relative plant traits presenting significant interactions during generative and vegetative phase experiments. (DOCX)

S1 Fig Experimental setup of the each experiment. (DOCX)

S2 Fig Model-based ancestry for each of the 192 accessions based on the 6259 biallielic markers from the 9K iSELECT SNP chip used to build the Q matrix. (DOCX)

S3 Fig Correlations diagram between selected phenotypic traits for vegetative. (a) and generative (b) experiments. Only significant correlation at p<0.05 are depicted. Refers to Table 1 for the acronyms.
(DOCX)

S4 Fig Correlations diagram between selected relative phenotypic traits for vegetative (a) and generative (b) experiments. Only significant correlation at p<0.01 are depicted. Refers to Table 1 for the acronyms.
(DOCX)
