# Supplementary material for: Physiological and morphological responses of different spring barley genotypes to water deficit and associated QTLs
Source: PLoS One. 2020 Aug 27;15(8):e0237834. doi: 10.1371/journal.pone.0237834 (PMC7451664; doi:10.1371/journal.pone.0237834)
Supplement: S1 Table — (DOCX) [file pone.0237834.s001.docx]

**S1 Table.** Details of experiment initiation and harvest dates

| Experiment | Sowing date | Germination date | Stress initiation | Final Harvest Date | Phase |
| --- | --- | --- | --- | --- | --- |
| 1 | June 7, 2012 | June 11, 2012 | June 25, 2012 | August 2, 2012 | vegetative |
| 2 | April 13, 2013 | April 25, 2013 | May 10, 2013 | August 9, 2013 | vegetative |
| 3 | June 20, 2013 | June 26, 2013 | July 23, 2013 | September 12, 2013 | generative |
| 4 | March 12, 2014 | March 26, 2014 | April 7, 2014 | July 1, 2014 | vegetative |
| 5 | June 18, 2014 | June 26, 2014 | July 17, 2014 | October 23, 2014 | generative |
| 6 | March 12, 2015 | March 29, 2015 | May 6, 2015 | July 1, 2015 | generative |
