# Supplementary material for: Physiological and morphological responses of different spring barley genotypes to water deficit and associated QTLs
Source: PLoS One. 2020 Aug 27;15(8):e0237834. doi: 10.1371/journal.pone.0237834 (PMC7451664; doi:10.1371/journal.pone.0237834)
Supplement: S2 Table — (DOCX) [file pone.0237834.s002.docx]

**S2 Table**. Analysis of variance for genotypic and treatment effects and their interactions on different absolute plant traits using a linear mixed model during the both the generative and vegetative stage experiments. Refer to Table 1 for all acronyms.

|  | **Generative stage experiment** | | | | **Vegetative stage experiment** | | | | **Generative stage experiment** | | | | **Vegetative stage experiment** | | | |
| --- | --- | --- | --- | --- | --- | --- | --- | --- | --- | --- | --- | --- | --- | --- | --- | --- |
| **Source** | **Trait** | **G** | **T** | **G x T** | **Trait** | **G** | **T** | **G x T** | **Trait** | **G** | **T** | **G x T** | **Trait** | **G** | **T** | **G x T** |
| **dF** | **DM** | 142 | 1 | 142 | **DM** | 100 | 1 | 100 | **DMLG** | 138 | 1 | 72 | **DMLG** | 99 | 1 | 98 |
| **F value** |  | 5.2 | 385 | 2.092 |  | 5.677 | 93.06 | 3.32 |  | 6.202 | 328.9 | 4.86 |  | 4.466 | 470.1 | 2.048 |
| **Sign.** |  | *** | ** | *** |  | *** | * | *** |  | *** | *** | *** |  | *** | ** | *** |
| **dF** | **DME** | 142 | 1 | 142 | **DME** | 97 | 1 | 65 | **DMLSms** | 142 | 1 | 142 | **DMLSms** | 100 | 1 | 98 |
| **F value** |  | 3.702 | 20.01 | 1.606 |  | 10.19 | 223.1 | 3.313 |  | 5.715 | 20.84 | 1.411 |  | 4.382 | 55.14 | 2.061 |
| **Sign.** |  | *** | * | *** |  | *** | *** | *** |  | *** | * | ** |  | *** | * | *** |
| **dF** | **DMEt** | 142 | 1 | 142 | **DMEt** | 73 | 1 | 27 | **DMEms** | 142 | 1 | 142 | **DMEms** | 97 | 1 | 61 |
| **F value** |  | 4.264 | 20.6 | 1.517 |  | 7.285 | 43.58 | 5.235 |  | 6.331 | 10.72 | 1.713 |  | 10.4 | 16.72 | 4.611 |
| **Sign.** |  | *** | * | *** |  | *** | ** | *** |  | *** | . | *** |  | *** | * | *** |
| **dF** | **DMLGt** | 137 | 1 | 70 | **DMLGt** | 97 | 1 | 92 | **DMSt** | 142 | 1 | 142 | **DMSt** | 99 | 1 | 97 |
| **F value** |  | 5.114 | 218.7 | 3.986 |  | 4.179 | 453.7 | 2.609 |  | 5.356 | 79.61 | 2.327 |  | 5.549 | 53.82 | 2.889 |
| **Sign.** |  | *** | *** | *** |  | *** | ** | *** |  | *** | * | *** |  | *** | * | *** |
| **dF** | **DMLGms** | 103 | 1 | 14 | **LAt** | 99 | 1 | 92 | **LAt** | 142 | 1 | 142 | **WU** | 100 | 1 | 100 |
| **F value** |  | 16.15 | 41.47 | 16.5 |  | 3.417 | 38.49 | 2.149 |  | 3.443 | 31.56 | 1.423 |  | 2.953 | 88.5 | 2.234 |
| **Sign.** |  | *** | *** | *** |  | *** | * | *** |  | *** | * | ** |  | *** | * | *** |
|  | **LAms** | 105 | 1 | 13 | **LAms** | 100 | 1 | 96 | **LA** | 136 | 1 | 70 | **TE** | 100 | 1 | 100 |
|  |  | 19.65 | 52.59 | 20.96 |  | 8.113 | 28.83 | 1.82 |  | 4.407 | 301.1 | 3.238 |  | 5.076 | 24.7 | 2.046 |
|  |  | *** | *** | *** |  | *** | * | *** |  | *** | *** | *** |  | *** | * | *** |
| **dF** | **NLG** | 138 | 1 | 71 | **NLG** | 100 | 1 | 98 | **Lt** | 100 | 1 | 98 | **ST** | 99 | 1 | 84 |
| **F value** |  | 4.769 | 46.98 | 4.448 |  | 5.15 | 154.2 | 2.002 |  | 5.16 | 358.4 | 1.948 |  | 0.830 | 69.3 | 1.60 |
| **Sign.** |  | *** | * | *** |  | *** | ** | *** |  | *** | ** | *** |  |  | *** | ** |
| **dF** | **NEt** | 142 | 1 | 142 | **NEt** | 76 | 1 | 25 | **OP** | 186 | 1 | 174 | **OP** | 199 | 1 | 198 |
| **F value** |  | 6.326 | 30.47 | 2.467 |  | 10.89 | 61.75 | 10.84 |  | 7.299 | 2082 | 4 |  | 4.294 | 3413 | 2.666 |
| **Sign.** |  | *** | * | *** |  | *** | *** | *** |  | *** | *** | *** |  | *** | *** | *** |
| **dF** | **BBCH** | 142 | 1 | 141 | **BBCH** | 100 | 1 | 100 | **PROL** | 202 | 1 | 195 | **PROL** | 200 | 1 | 199 |
| **F value** |  | 2.617 | 3.412 | 1.499 |  | 5.638 | 111.5 | 1.42 |  | 2.216 | 285.4 | 1.733 |  | 2.91 | 240.8 | 2.904 |
| **Sign.** |  | *** | . | *** |  | *** | *** | * |  | *** | ** | *** |  | *** | ** | *** |
| **dF** | **Nt** | 142 | 1 | 142 | **Nt** | 100 | 1 | 98 | **RWC** | 198 | 1 | 197 | **RWC** | 190 | 1 | 188 |
| **F value** |  | 3.477 | 13.61 | 1.716 |  | 4.464 | 55.75 | 3.416 |  | 3.838 | 63.83 | 2.541 |  | 6.246 | 1718 | 3.561 |
| **Sign.** |  | *** | . | *** |  | *** | * | *** |  | *** | * | *** |  | *** | *** | *** |
| **dF** | **HI** | 142 | 1 | 142 | **HI** | 97 | 1 | 65 | **SLAt** | 135 | 1 | 67 | **SLAt** | 97 | 1 | 90 |
| **F value** |  | 4.011 | 1E-05 | 0.916 |  | 13.41 | 0.098 | 2.621 |  | 11.61 | 319.8 | 5.756 |  | 5.745 | 10.52 | 2.842 |
| **Sign.** |  | *** |  |  |  | *** |  | *** |  |  |  |  |  |  |  |  |
| **dF** | **Lms** | 142 | 1 | 142 | **Lms** | 100 | 1 | 100 | **SLAms** | 103 | 1 | 13 | **SLAms** | 99 | 1 | 94 |
| **F value** |  | 6.929 | 5.881 | 0.772 |  | 6.603 | 358.2 | 3.159 |  | 22.88 | 59.8 | 18.48 |  | 4.408 | 19.05 | 2.024 |
| **Sign.** |  | *** |  |  |  | *** | ** | *** |  | *** | *** | *** |  | *** | * | *** |
| **dF** | **LFms** | 96 | 1 | 14 | **LFms** | 99 | 1 | 87 | **SLA** | 136 | 1 | 69 | **SLA** | 99 | 1 | 98 |
| **F value** |  | 21.3 | 31.37 | 19.64 |  | 14.19 | 33.03 | 1.996 |  | 11.17 | 249.5 | 5.38 |  | 3.654 | 18.97 | 1.933 |
| **Sign.** |  | *** | *** | *** |  | *** | * | *** |  | *** | *** | *** |  | *** | * | *** |

Refer to Table 1 for all acronyms. G: Genotype, T: Treatment and GxT: genotype x treatment interaction; Sign.: significance codes: '***' 0.001; '**' 0.01; '*' 0.05; '.' 0.1 ;
