# Supplementary material for: Physiological and morphological responses of different spring barley genotypes to water deficit and associated QTLs
Source: PLoS One. 2020 Aug 27;15(8):e0237834. doi: 10.1371/journal.pone.0237834 (PMC7451664; doi:10.1371/journal.pone.0237834)
Supplement: S3 Table — (DOCX) [file pone.0237834.s003.docx]

**S3 Table.**  Analysis of variance (ANOVA) of relative plant traits presenting significant interactions during generative and vegetative phase experiments.

|  | **Generative phase experiment** | | | **Vegetative stage experiment** | | | **Generative stage experiment** | | | **Vegetative stage experiment** | | |
| --- | --- | --- | --- | --- | --- | --- | --- | --- | --- | --- | --- | --- |
| **Source** | **Trait** | **Year** | **G** | **Trait** | **Year** | **G** | **Trait** | **Year** | **G** | **Trait** | **Year** | **G** |
| **dF** | **OA** | 2 | 180 | **OA** | 2 | 185 | **rHI** | 2 | 142 | **rHI** | 2 | 62 |
| **Sign.** |  | *** | *** |  | *** | *** |  | *** |  |  |  | *** |
| **dF** | **rWSC** | 2 | 197 | **rTE** | 2 | 100 | **rNLG** | 2 | 64 | **rNLG** | 2 | 98 |
| **Sign.** |  | *** | *** |  |  | *** |  |  | *** |  |  | *** |
| **dF** | **rLALG** | 2 | 63 | **WU** | 2 | 100 | **rNEt** | 2 | 142 | **rNEt** | 2 | 25 |
| **Sign.** |  | *** | *** |  | *** | *** |  |  | *** |  | . | *** |
| **dF** | **rRWC** | 2 | 197 | **rRWC** | 2 | 187 | **rNt** | 2 | 142 | **rNt** | 2 | 98 |
| **Sign.** |  | *** | *** |  | *** | *** |  |  | *** |  |  | *** |
| **dF** | **rOP** | 2 | 173 | **rOP** | 2 | 198 | **rLAt** | 2 | 141 | **rLAt** | 2 | 91 |
| **Sign.** |  |  | *** |  |  | *** |  |  | *** |  |  | *** |
| **dF** | **rPROL** | 2 | 195 | **rPROL** | 2 | 199 | **rDMLGms** | 2 | 65 | **rDMLGms** | 2 | 91 |
| **Sign.** |  | *** | *** |  | *** | *** |  | . | *** |  | ** | *** |
| **dF** | **rDMLSt** | 2 | 142 | **rDMLSt** | 2 | 94 | **rDMLG** | 2 | 98 | **DMEms** | 2 | 142 |
| **Sign.** |  | . | *** |  | . | *** |  | * | *** |  | * | *** |
| **dF** | **rDMSms** | 2 | 142 | **rDMSms** | 2 | 99 | **rBBCH** | 2 | 141 | **rBBCH** | 2 | 100 |
| **Sign.** |  | *** | *** |  |  | *** |  |  | *** |  |  | *** |
| **dF** | **rDME** | 2 | 142 | **rDME** | 2 | 62 | **rLFms** | 2 | 14 | **rLFms** | 2 | 83 |
| **Sign.** |  | *** | *** |  |  | *** |  |  | *** |  | . | *** |
| **dF** | **rDMEt** | 2 | 142 | **rDMEt** | 2 | 26 | **rLms** | 2 | 142 | **rLms** | 2 | 100 |
| **Sign.** |  |  | *** |  |  | *** |  |  | *** |  |  | *** |
| **dF** | **rDMLGt** | 2 | 63 | **rDMLGt** | 2 | 92 | **rLt** | 2 | 142 | **rLt** | 2 | 98 |
| **Sign.** |  | ** | * |  |  | *** |  |  | *** |  |  | *** |
| **dF** | **rDMSt** | 2 | 142 | **rDMSt** | 2 | 97 | **rSLA** | 2 | 63 | **rSLA** | 2 | 97 |
| **Sign.** |  |  | *** |  |  | *** |  | * | *** |  | * | *** |
| **dF** | **DMLSms** | 2 | 142 | **DMLSms** | 2 | 98 | **rSLAms** | 2 | 13 | **rSLAms** | 2 | 91 |
| **Sign.** |  |  | *** |  | ** | *** |  |  | *** |  | . | *** |
| **dF** | **rDM** | 2 | 142 | **rDM** | 2 | 100 | **rSLAt** | 2 | 61 | **rSLAt** | 2 | 90 |
| **Sign.** |  | * | *** |  |  | *** |  | * | *** |  |  | *** |

Refer to Table 1 for all acronyms. G: Genotype; Sign.: significance codes: '***' 0.001; '**' 0.01; '*' 0.05; '.' 0.1 ;
