# Supplementary figures and images for: Physiological and morphological responses of different spring barley genotypes to water deficit and associated QTLs
Source: PLoS One. 2020 Aug 27;15(8):e0237834. doi: 10.1371/journal.pone.0237834 (PMC7451664; doi:10.1371/journal.pone.0237834)

**S1 Fig. Experimental setup of the each experiment.**


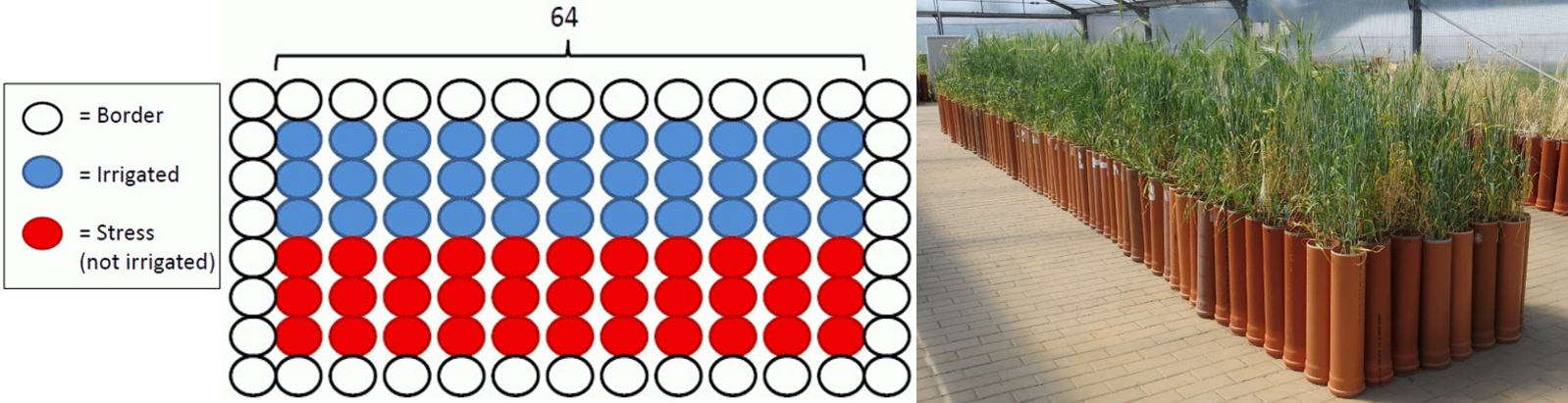

Supplement: S1 Fig — (DOCX) [file pone.0237834.s004.docx]
