# Supplementary material for: Physiological and morphological responses of different spring barley genotypes to water deficit and associated QTLs
Source: PLoS One. 2020 Aug 27;15(8):e0237834. doi: 10.1371/journal.pone.0237834 (PMC7451664; doi:10.1371/journal.pone.0237834)

**S2 Fig. Model-based ancestry for each of the 192 accessions based on the 6259 biallielic markers from the 9K iSELECT SNP chip used to build the Q matrix**.


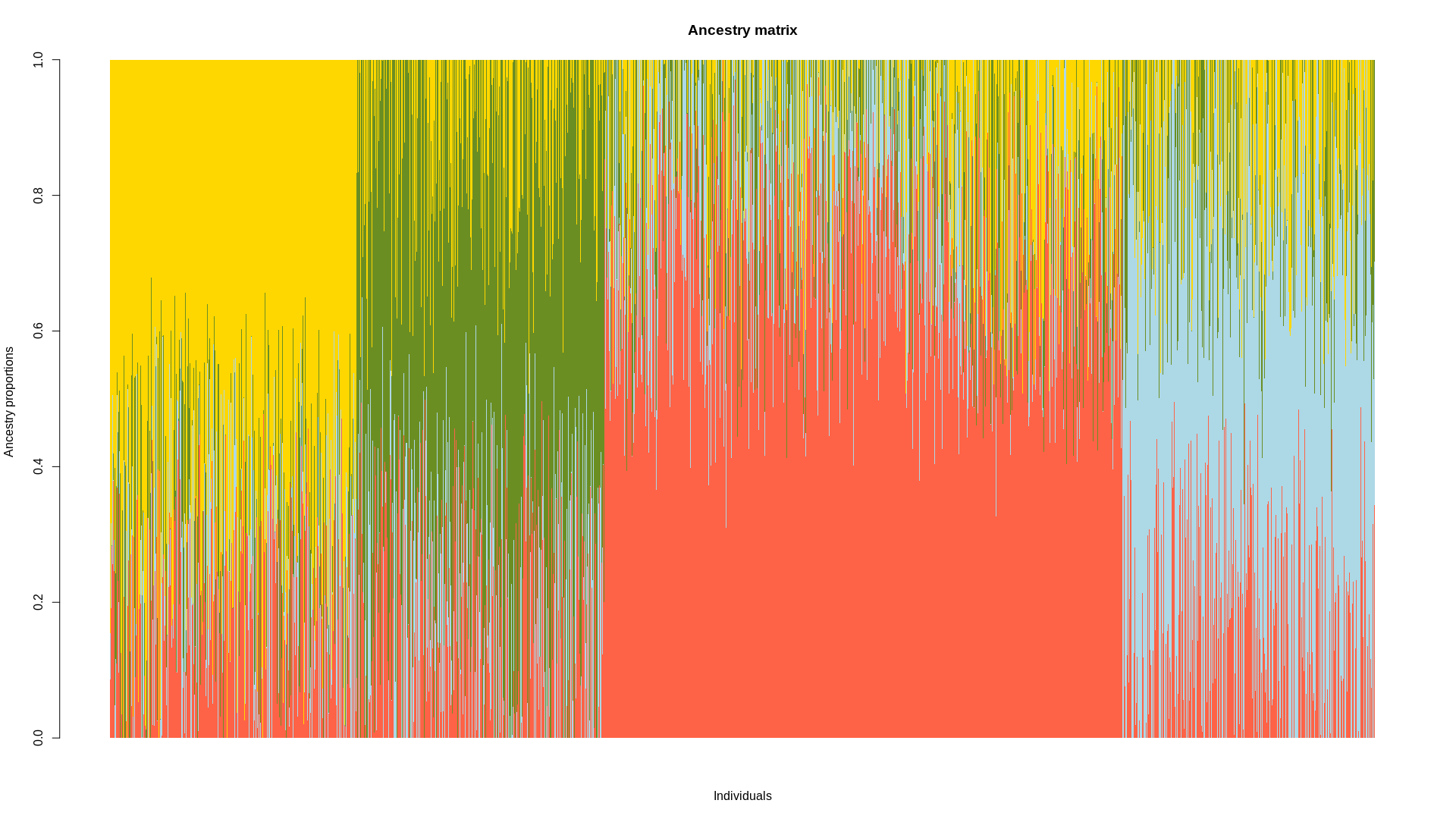

Supplement: S2 Fig — (DOCX) [file pone.0237834.s005.docx]
