# Supplementary material for: Physiological and morphological responses of different spring barley genotypes to water deficit and associated QTLs
Source: PLoS One. 2020 Aug 27;15(8):e0237834. doi: 10.1371/journal.pone.0237834 (PMC7451664; doi:10.1371/journal.pone.0237834)
Supplement: S4 Fig — Only significant correlation at p<0.01 are depicted. Refers to Table 1 for the acronyms. (DOCX) [file pone.0237834.s007.docx]

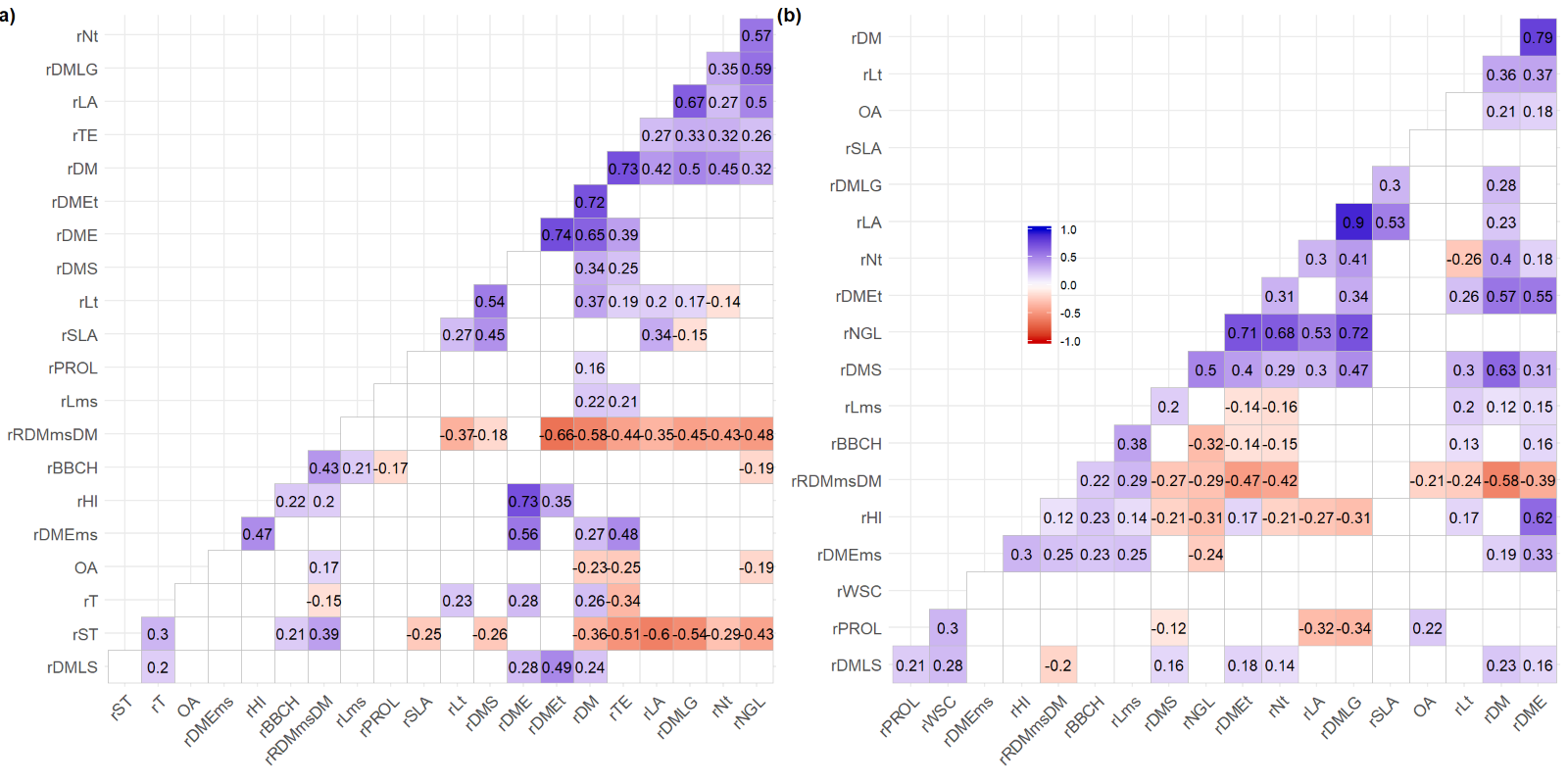


**S4 Fig. Correlations diagram between selected relative phenotypic traits for vegetative** (a) and generative (b) experiments. Only significant correlation at p<0.01 are depicted. Refers to table 1 for the acronyms
